# Screening the Surface Structure-Dependent Action of a Benzotriazole Derivative on Copper Electrochemistry in a Triple-Phase Nanoscale Environment

**DOI:** 10.1021/acs.jpcc.2c04494

**Published:** 2022-08-29

**Authors:** Enrico Daviddi, Viacheslav Shkirskiy, Paul M. Kirkman, Mathew P. Robin, Cameron L. Bentley, Patrick R. Unwin

**Affiliations:** † Department of Chemistry, University of Warwick, Coventry CV4 7AL, U.K.; ‡ Université Paris Cité, ITODYS, CNRS, Paris F-75006, France; § Lubrizol LTD, Nether Ln, Hazelwood DE56 4AN, U.K.; ∥ School of Chemistry, Monash University, Clayton, Victoria 3800, Australia

## Abstract

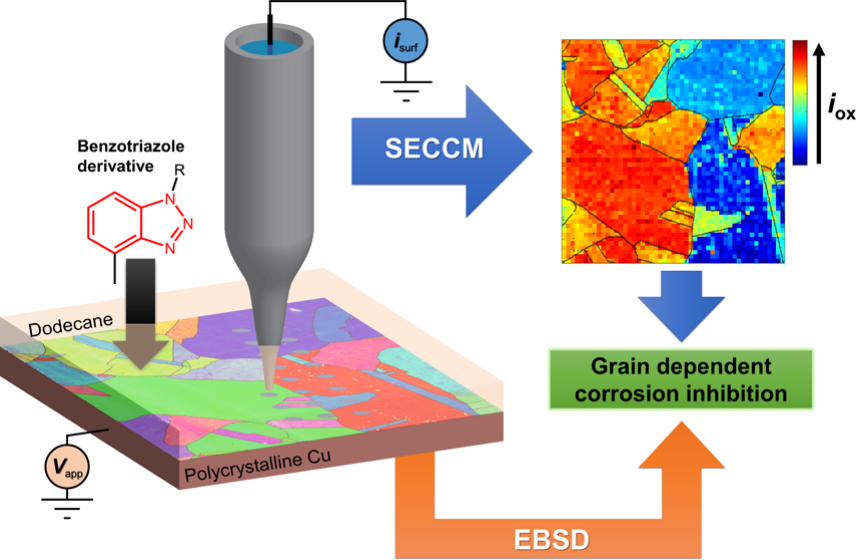

Copper (Cu) corrosion is a compelling problem in the automotive sector and in oil refinery and transport, where it is mainly caused by the action of acidic aqueous droplets dispersed in an oil phase. Corrosion inhibitors, such as benzotriazole (BTAH) and its derivatives, are widely used to limit such corrosion processes. The efficacy of corrosion inhibitors is expected to be dependent on the surface crystallography of metals exposed to the corrosion environment. Yet, studies of the effect of additives at the local level of the surface crystallographic structure of polycrystalline metals are challenging, particularly lacking for the triple-phase corrosion problem (metal/aqueous/oil). To address this issue, scanning electrochemical cell microscopy (SECCM), is used in an acidic nanodroplet meniscus|oil layer|polycrystalline Cu configuration to explore the grain-dependent influence of an oil soluble BTAH derivative (BTA-R) on Cu electrochemistry within the confines of a local aqueous nanoprobe. Electrochemical maps, collected in the voltammetric mode at an array of >1000 points across the Cu surface, reveal both cathodic (mainly the oxygen reduction reaction) and anodic (Cu electrooxidation) processes, of relevance to corrosion, as a function of the local crystallographic structure, deduced with co-located electron backscatter diffraction (EBSD). BTA-R is active on the whole spectrum of crystallographic orientations analyzed, but there is a complex grain-dependent action, distinct for oxygen reduction and Cu oxidation. The methodology pinpoints the surface structural motifs that facilitate corrosion-related processes and where BTA-R works most efficiently. Combined SECCM–EBSD provides a detailed screen of a spectrum of surface sites, and the results should inform future modeling studies, ultimately contributing to a better inhibitor design.

## Introduction

Owing to its high thermal and electrical conductivity, copper (Cu) is widely used in many sectors of modern industry and everyday life.^1^ As such, Cu corrosion has significant socioeconomic impacts, and ameliorating this issue has inspired fundamental and applied research, with most attention concentrated in marine environments (i.e., concentrated aqueous chloride solutions).^2^ However, Cu is also widely used in the automotive and oil refinery industries, where the active corrosion agents are typically oil-soluble acids (e.g., sulfonic and/or carboxylic acids and their salts) which may partition into the small amount of water that contaminates all oil products.^3,4^ The corrosion-action of water-soluble inorganic acids is of great importance in the automotive industry because waste products (e.g., carbon, nitrogen, and sulfur oxides) originating from the combustion chamber form acidic nanodroplets within the oil phase, which can induce localized corrosion upon contact with the metallic (e.g., Cu) surfaces of the engine.^5,6^

**Figure 1:**
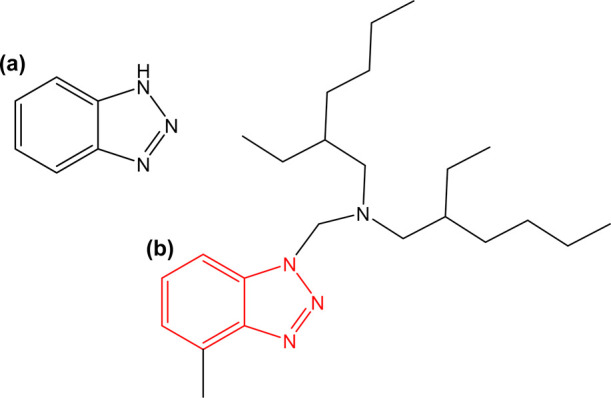
Chemical structures of (a) BTAH and (b) *N*,*N*-bis-(2-ethylhexyl)-4-methyl-1*H*-benzotriazole-1-methanamine (BTA-R), the commercial oil soluble derivative considered in this contribution. The red structure in (b) highlights the part of the molecule in common between (a,b).

To control and minimize the effect of these corroding agents, many different corrosion inhibitors can be used. The most studied, and arguably the most important, corrosion inhibitor for Cu surfaces is 1,2,3-benzotriazole (BTAH), which has been known since the late 1940s and applied as a corrosion suppressor since the 1960s.^7^ The action of BTAH in suppressing and controlling Cu degradation is mainly due its strong interaction with the metal surface, predominantly through the nitrogen atoms in the heterocyclic ring (especially when it is in the deprotonated form, BTA^–^), as well as the π electrons of the aromatic ring (Figure 1a). BTAH adsorption results in the formation of supramolecular structures at the Cu surface, which effectively act as a physical barrier to the surrounding environment.^8−10^ Since its inception, many BTAH derivatives have been synthesized to adapt its use to different conditions,^11−13^ for example, oil-soluble derivatives are used in the oil and automotive industries to minimize the aforementioned Cu degradation.^14^

The local corrosion-related properties of metals, including the inhibitory effects of corrosion inhibitors such as BTAH, are known to depend strongly on microscopic surface properties, such as composition, structure, and morphology.^15−18^ Recent experimental and theoretical investigations by this group on Cu,^19^ Zn,^20^ and low carbon steel,^21−23^ have demonstrated strong grain (and in some cases, grain boundary)-dependent corrosion properties, established across the whole spectrum of surface crystallographic orientations. This holistic view of corrosion resistance/susceptibility was brought forth through the application of scanning electrochemical cell microscopy (SECCM) in tandem with complementary, co-located surface analysis techniques, specifically electron backscatter diffraction (EBSD) and scanning electron microscopy (SEM). SECCM is the latest generation of electrochemical microcell techniques, employing a fine laser-pulled pipet (typically 30 nm to 1 μm diameter) and piezoelectric positioners to rapidly translate a small droplet cell across an electrode surface to perform high-resolution electrochemical mapping in an automated fashion.^24,25^

In a very recent study, SECCM was used for electrochemical measurements across a polycrystalline Cu surface immersed in a model mineral oil (dodecane), effectively simulating the aqueous nanodroplet/oil/metal interface relevant to automotive engine corrosion. Both anodic and cathodic processes in a nanodroplet acidic environment (sulfuric acid, pH ≈ 2) were examined, revealing a wide and complex grain dependency, not predictable simply through the combination of the constituent low-index grain ({001}, {011}, and {111}) response(s).^19,26^ A natural development of this work is to study the grain-specific action of inhibitors. For BTAH, hitherto, the main focus has been well-defined, low-index crystallographic planes of Cu using macroscopic single-crystal electrodes (i.e., {001}, {011}, and {111}).^27−29^

This work aims to exploit the power of co-located SECCM–EBSD for understanding the structure-dependent efficacy of an oil soluble BTAH derivative (commercial name Irgamet 39, Figure 1b, named BTA-R for brevity), by comparing local voltammetric results collected in the absence and presence of BTA-R, dissolved in the oil phase. As in previous work,^19^ the SECCM tip contains the aggressive acidic medium, and so the experiment closely mimics the nanodroplet/oil/metal interface that is of practical relevance, with the bonus that it is possible to study the action of a completely *oil-soluble* inhibitor on local *aqueous* corrosion, which would be difficult to achieve with other electrochemical techniques. By studying a polycrystalline sample on a small scale, it is possible to screen a wide range of surface crystallography, including surfaces that have not been studied previously. Thus, this work demonstrates how SECCM methodology can elucidate the grain-dependent action of BTA-R on Cu surfaces under industrially relevant conditions, closing the knowledge gap between electrochemical surface science and practical application. Furthermore, the study of electrochemical reactions that happen at a triple-phase interface,^30,31^ where electron transfer is often coupled with phase transfer, is of great interest in different fields of application of electrochemistry, and this work provides further tools to perform such studies at the nanoscale.

## Methods

### Chemical Reagents and Electrode Materials

Sulfuric acid (H_2_SO_4_, Sigma-Aldrich, Germany, 96%), potassium chloride (KCl, Honeywell, Germany, 99.5%), *n*-dodecane (C_12_H_26_, Sigma-Aldrich, Germany, 99%), and BTA-R (provided by Lubrizol Ltd, available as Irgamet 39, BASF, Germany) were employed as supplied. All aqueous solutions were prepared with ultrapure deionized water (resistivity = 18.2 MΩ.cm at 25 °C).

Cu substrates were prepared by cutting a 3 mm thick foil (Goodfellow, U.K., 99.95%) into rectangles of about 1 cm × 2 cm, which were annealed in a furnace at 800 °C under an argon atmosphere for 12 h. The substrates were then mounted in a conductive carbon support (KonductoMet, Buehler, U.S.A.) with a SimpliMet 3000 mounting press, with a hot mounting procedure at 190 °C and then polished to a mirror finish with a Buehler AutoMet 300 Pro polishing machine. The mirror polishing was performed on polishing pads with aqueous polishing suspension (Buehler, U.S.A.) as follows: (1) 9 μm MetaDi Supreme Diamond suspension on a TexMet C polishing pad; (2) 3 μm MetaDi Supreme Diamond suspension on a Verdutext polishing pad; and (3) 0.02–0.06 μm Master-mMet Colloidal Silica suspension on a ChemoMet polishing pad. Following the polishing procedure, the sample was washed in deionized water and isopropanol and blown dried with warm air. Before the SECCM experiment, a reservoir was created around the Cu surface to contain the mineral oil solution, by erecting a 2 mm high and 3 mm thick barrier of chemically resistant epoxy resin over the edge of the carbon mounting.^19^

The Ag/AgCl quasi-reference counter electrode (QRCE) was prepared by the anodization of an annealed silver wire (0.25 mm diameter, Goodfellow, U.K., 99.99%) in a saturated KCl solution for *ca.* 2 min. The QRCE potential was then calibrated after each SECCM scan by measuring the open circuit potential in the solution of interest (10 mM H_2_SO_4_) versus a commercial 3.4 M Ag/AgCl electrode (ET072, eDAQ, Australia), which has a standard potential of +0.205 V versus the standard hydrogen electrode.^32^ The measured potential for the QRCE was within the range of *ca.* +0.21 to +0.26 V versus Ag/AgCl, comparable to previous reports^33^ and stable on the experimental timescale (i.e., 1–2 h).

### Scanning Electrochemical Cell Microscopy

The SECCM experiments were carried out on a home-built scanning electrochemical probe microscopy system. All data acquisition and instrumental control was carried out with an FPGA card (PCIe-7852R) controlled by a LabVIEW 2016 (National Instruments, U.S.A.) interface running the Warwick Electrochemical Scanning Probe Microscopy software (WEC-SPM, available for download at www.warwick.ac.uk/electrochemistry). A home-built electrometer (with fA to nA sensitivity) was used and the applied potential and positional control came from the FPGA card. For fine movements (approaches and scanning) between the sample and the probe, piezoelectric positioners (for *x*–*y* positioning: P-733.2, 100 × 100 μm^2^; for *z* positioning P-753.2, 38 μm, Physik Instrumente, Germany) were employed, and *x*–*y*–*z* coarse micropositioners (M-461-XYZ-M, Newport, U.S.A.) were used for preliminary positioning.^34,35^

The SECCM probes were fabricated from a borosilicate glass capillary (GC120F-10, Harvard Apparatus, U.S.A.) with a commercial CO_2_ laser puller (P-2000, Sutter Instruments, U.S.A), with pulling parameters reported in Supporting Information, Section S.1. For each borosilicate capillary, two closely similar sharp point nanopipets were obtained, each with an ending aperture of diameter ≈400 nm (verified through scanning transmission electron microscopy, not shown). After pulling, the nanopipets were filled with electrolyte (10 mM H_2_SO_4_), topped with a thin silicone oil layer (*ca.* 1–2 mm thick), to minimize back-evaporation during prolonged SECCM scanning.^36^ After filling, an Ag/AgCl QRCE was inserted from the back into the probe, reaching roughly 3 cm from the tip end.^33^

The Cu substrate was mounted on the *x*–*y* piezoelectric positioners, while the prepared SECCM probe was mounted on the *z* piezoelectric positioner and moved to the initial position of the scan, roughly 20 μm above the Cu surface, with the *x*–*y*–*z* coarse micropositioners. The process was monitored with an optical camera (PLB776U camera equipped with a 6× lens, Pixelink, Canada). Then, the substrate (and consequentially the probe tip) was covered with ≈2 mm thick layer of a mineral oil solution, consisting of either pure dodecane or a 100 ppm (w/w) solution of BTA-R in dodecane. The entire set up (i.e., SECCM probe and immersed Cu substrate) was exposed to the ambient air, and the BTA-R solution, when employed, was allowed to settle on the surface for about an hour prior to measurement. When measurements were performed in the absence of O_2_ (i.e., de-aerated conditions), the tip of the SECCM probe and the immersed Cu substrate were placed in a custom-built environmental cell,^26,37^ which was mounted on the *x*–*y* piezoelectric positioner and purged with high-purity Ar at a flow rate of 80 mL min^–1^ for about 1 h prior to use, which was also maintained for the duration of all SECCM experiments. The entire SECCM configuration was placed on a passive mechanical vibration isolator platform (Minus K Technology, U.S.A.), located in an aluminum Faraday cage, equipped with heat sinks and acoustic foam to minimize mechanical vibration, electrical noise, and thermal drift.

SECCM experiments were carried out in the hopping mode, applying potential-controlled electrochemical techniques, in particular both stationary potential pulses (chronoamperometry) and potential sweeps (cyclic voltammetry), as previously described.^20,38−40^ The nanopipet probe, always immersed in the mineral oil layer, was approached (with positional feedback) to the Cu substrate until meniscus-surface contact was made (the glass portion of the probe never made direct contact with the surface). During the approach, an initial potential (*E*_i_) of −0.7 V versus Ag/AgCl QRCE (*ca.* −0.45 V vs Ag/AgCl 3.4 M KCl) was applied at the Cu substrate, and positional feedback was achieved by monitoring the current flowing between the Cu working electrode and Ag/AgCl QRCE (surface current, *i*_surf_), where the *z*-approach was halted upon the detection of an absolute current change >1.5 pA, indicating that a two-electrode electrochemical cell had formed between the substrate working electrode and the tip QRCE. Unless otherwise stated, the *E* scale is always referred versus the Ag/AgCl (3.4 M KCl) reference and not versus the QRCE.

After landing, the potential was held at *E*_i_ for 0.25 s, before being swept linearly in the positive direction at voltammetric scan rate (υ) = 1 V s^–1^. After reaching the final potential (*E*_f_) values of *ca.* +0.24 and +0.40 V versus Ag/AgCl under aerated and de-aerated conditions, respectively, the direction of the linear potential sweep was reversed back toward *E*_i_ to produce a cyclic voltammogram (CV). After completing the CV, the probe was retracted and moved to the next point of the scan, with such operation repeated for each of a series of predefined points of a rectangular grid. The spatially resolved CVs were arranged to create potential-time-dependent two-dimensional maps of the recorded current *i*_surf_, effectively creating high-resolution electrochemical movies, as previously reported.^36,38^ Note that although a full CV was recorded for each point, only the forward sweep is presented as a linear sweep voltammogram (LSV) in the main text. The reverse process ensured that a significant proportion of Cu anodically dissolved in the forward sweep was redeposited before the tip moved to the next spot, with the remaining Cu^2+^ diffusing to bulk solution of the probe.

During scanning, the nanopipet was approached at a speed of 3 μm s^–1^, retracted at 10 μm s^–1^ (for a distance of 2 μm), and moved laterally at a speed of 20 μm s^–1^ between each point. The hopping distance (i.e., the distance between two consecutive single measurements) was set at 2 μm to avoid overlapping of the areas wetted by the meniscus, keeping each CV measurement independent. The voltage was applied at the QRCE and *i*_surf_ was measured with the electrometer mentioned above, with data points acquired every 4 μs, and 128 points averaged, to give a data acquisition rate of 4 × (128 + 1) = 516 μs per point (one extra iteration was used to transfer the data to the host computer). SECCM data were analyzed with custom scripts in MATLAB R2020b (MathWorks, U.S.A.) with the data plotted with OriginPro 2020 64 bit (9.60, OriginLab, U.S.A.) and MATLAB R2020b (for the movies and all the 2D electrochemical maps) software packages. All electrochemical images and movies were plotted without any data interpolation. When presented, the median curves were calculated by taking the median of all LSVs measured in a given scan area (i.e., roughly 5400 different points, except for the comparison of curves measured in de-aerated conditions, which are the median of a few tens of point measurements).

### Surface Characterization

SEM and EBSD were performed on a JEOL JSM-7800F FEG-SEM (Zeiss, Germany), equipped with a Nordlys EBSD detector (Oxford Instruments, U.K.). SEM images were taken with an InLens detector and an acceleration voltage of 5 keV and employed to estimate the contact area of the SECCM meniscus cell (i.e., droplet footprint) on the polycrystalline Cu surface after the scan, with typical values being ≈600 nm diameter (area ≈ 3 × 10^–9^ cm^2^). This value was deduced by averaging 10 wetted areas from different points within each scan area. There was little detectable difference within a scan, whereas there was a slight difference between scans with and without BTA-R (*vide infra*). EBSD mapping was carried out after each SECCM scan with an acceleration voltage of 20 keV and the sample tilted 70° to the detector. Data were processed with HKL CHANNEL5 software (Tango, Oxford Instruments, U.K.) to extract inverse pole figure (IPFz) images and the average grain orientations characterizing the areas scanned with SECCM.

## Results and Discussion

### Effect of O_2_ on Cu Voltammetry

**Figure 2:**
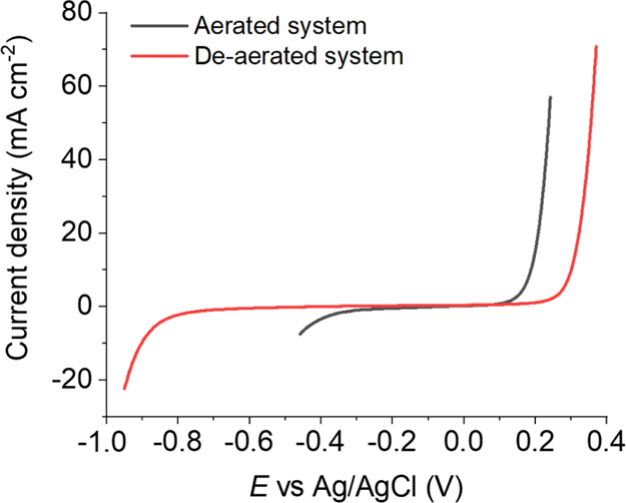
LSVs obtained in the SECCM triple-phase setup, at voltammetric sweep rate υ = 1 V s^–1^, on a polycrystalline Cu surface, in either aerated (gray curve) or de-aerated conditions (red curve). Each curve was preceded by a landing period of 0.25 s at the start potential (respectively −0.44 and −0.95 V). The presented curves are the median taken from 5426 (for the aerated case) and 102 (for the de-aerated case) individual measurements. These measurements were obtained in the SECCM configuration with a nanopipet probe containing 0.01 M H_2_SO_4._

Cu corrosion is the result of a complex interplay between various cathodic and anodic processes and is particularly influenced by the presence of O_2_ in the system. In the three-phase nanodroplet cell setup herein, O_2_ partitions across the oil/nanodroplet meniscus interface, with the oil layer (where the oxygen solubility is higher) effectively serving as a reservoir. To better understand which electrochemical reactions were affected by the action of the corrosion inhibitor, BTA-R, the voltammetric behavior of Cu was first investigated in both aerated and de-aerated environments (i.e., in an argon purged cell); median SECCM LSV curves are presented in Figure 2.

Each LSV measurement was preceded by a 0.25 s landing pulse at the initial potential, which served to electrochemically reduce the native surface passive layers (mainly oxides layers, due to 1 day elapsing between the polishing and the SECCM measurements).^41−43^ Although the charge passed during the landing period was not sufficient to completely reduce the oxide layer on the surface (possibly leading to a Cu|oxide|Cu sandwich structure), oxide reduction was quantitatively grain-dependent. However, this only had a mild influence (correlation) with the median LSV curves, considered herein. More detailed discussion on the oxide reduction during the landing period and its relation with grain structure and the Cu electro-oxidation current can be found in the Supporting Information, Section S.2. During the LSV measurements, the potential was swept anodically at a voltammetric scan rate (υ) = 1 V s^–1^ to characterize the electro-dissolution of the Cu surface. A cathodic current wave is measured at the start potential, attributed to the oxygen reduction reaction (ORR), driven by the high abundance of O_2_ through continuous exchange with the oil phase,^19^ highlighted also by the comparison with a similar measurement on a macrodisk electrode, where the cathodic current at −0.45 V is negligible (see Supporting Information, Section S.3, Figure S3). In contrast, under de-aerated conditions, the potential for the cathodic process is shifted by *ca.* −0.450 V, as the main cathodic process is the hydrogen evolution reaction. At the most positive potentials, an anodic current flows and the onset of the wave is shifted by *ca.* +0.1 V under de-aerated compared to aerated conditions because Cu electro-dissolution involves intermediates [such as Cu(I) in various forms on the surface] which are oxidized by O_2_.^44−46^ The anodic shift of the wave under de-aerated conditions, compared to aerated was not influenced by the meniscus landing and start potential (Supporting Information, Figure S4). The remaining studies consider aerated conditions as this is most relevant for the practical corrosion process.

### Effect of BTA-R on Cu Voltammetry

**Figure 3:**
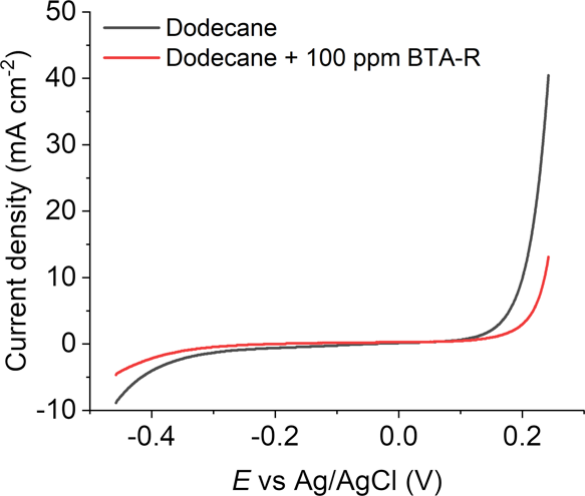
Comparison of the median LSV curves without (gray line, reproduced from Figure 2) and with (red line) the addition of BTA-R to the oil layer (100 ppm). A triple-phase SECCM setup was used with naturally aerated conditions, averaging 5426 individual measurements in each case.

Figure 3 compares the electrochemistry of Cu under a layer of dodecane in the absence (gray line) and the presence (red line) of BTA-R (100 ppm weight, dissolved in the oil phase) under aerated conditions. Clearly, the inhibitor diminishes the magnitude of both the cathodic and anodic currents. Note that the addition of BTA-R in the oil phase decreases the wetting areas of the SECCM meniscus, as discussed in Supporting Information, Section S.3. To compare SECCM data with and without BTA-R, current density is used throughout. Although Figures 2 and 3 show median voltammetric responses, the SECCM provides spatially resolved voltammetry that can be represented as electrochemical activity movies. These are Movies S1 and S2 for the uninhibited case and S3 and S4 for the inhibited case (Supporting Information, Section S.4). As explored below, such movies can be readily correlated to co-located structural information (i.e., crystallographic orientation from EBSD) to resolve nanoscale structure–activity directly and unambiguously.^38,47−49^ This inhibitory action is likely to be affected by the surface structure, in particular the crystallographic orientation, as BTAH and its derivatives form different structures on different grains of Cu.^28,29,50^

### Overview of the Grain-Dependent Processes

**Figure 4:**
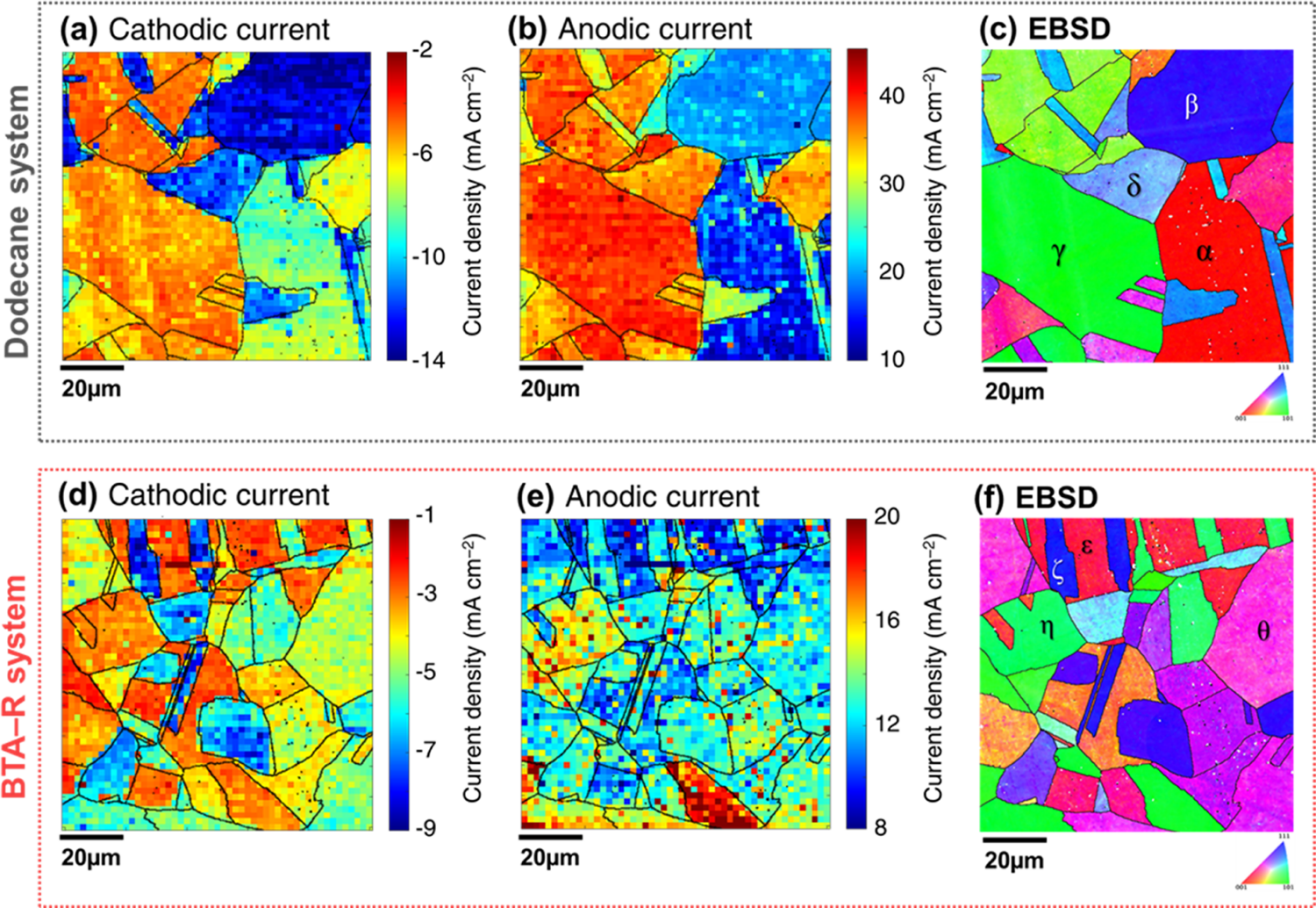
Electrochemical images extracted from Movies S1 and S3 with respective crystallographic orientation maps. (a,b) Single frames of Movie S1 showing the current density recorded at (a) *E* = −0.44 V and (b) *E* = +0.24 V with pure dodecane as the oil phase. (c) Co-located crystallographic orientation map, obtained with EBSD after SECCM. (d,e) Single frames of Movie S3 showing the current density recorded at (d) *E* = −0.44 V and (e) *E* = +0.24 V with a solution of BTA-R 100 ppm in dodecane as the oil phase. (f) Co-located crystallographic orientation map, obtained with EBSD after SECCM. All electrochemical measurements were conducted with a 10 mM H_2_SO_4_ solution in the probe. For clarity, the grain boundaries identified from the crystallographic orientation map were overlaid on the electrochemical images. Relevant grains are marked with the letters α–θ on the EBSD maps.

Figure 4a,b shows frames from Movie S1, measured in the absence of BTA-R, at the cathodic and the anodic limits of the voltammetric sweep, −0.45 and +0.24 V, respectively. Comparing these equipotential images with the co-located crystallographic orientation map (Figure 4c) reveals the grain dependency of the cathodic and anodic processes. The cathodic and anodic current density is depressed when the inhibitor is present, as shown in Figure 4d,e, respectively, but the grain-dependent behavior is still observed, with the co-located crystallographic orientation map shown in Figure 4f.

**Figure 5:**
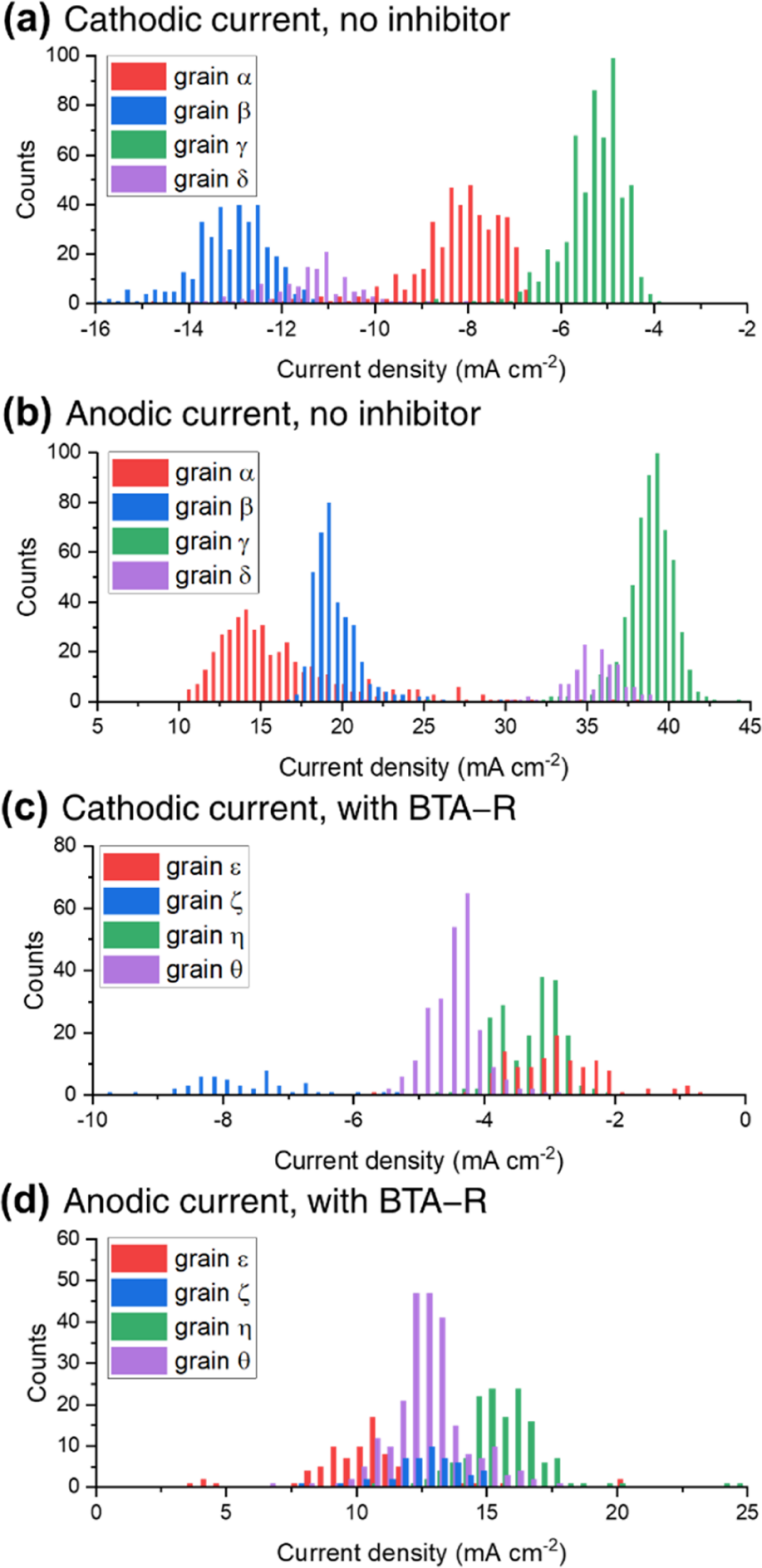
Distribution of current densities on selected grains, indicated in Figure 4c,f: (a,b) without the inhibitor at (a) *E* = −0.44 V and (b) *E* = +0.24 V, extracted, respectively, from Figure 4a,b for grains α–δ; (c,d) with BTA-R at (c) *E* = −0.44 V and (d) *E* = +0.24 V, extracted, respectively, from Figure 4d,e and grains ε–θ.

To highlight the spatial variability of electrochemical activity, current density distributions were constructed for the grains indicated as α–δ in Figure 4c and ε–θ in Figure 4f. These specific grains were selected to be representative of “low-index” [{001} (with α and ε), {111} (with β and ε), and {011} (with γ and η)] and “high-index” (with δ and θ) grains (specific orientation and electrochemical data of the selected grains are in Supporting Information, Section S.6). Note that grains δ and θ cannot be directly compared because they possess dramatically different orientations. Figure 5a,b shows the distributions of current density (extracted from the images in Figure 4a,b) within grains α–δ, respectively, for the cathodic (Figure 5a) and anodic (Figure 5b) processes. Despite the variability of current densities recorded within each grain (standard deviations of 2 and 4 mA cm^–2^ for the cathodic and anodic processes, respectively), a clear order of activity can be identified, with β > δ > α > γ for the cathodic process (i.e., the ORR) and γ > δ ≫ β > α for the anodic process (i.e., Cu dissolution), clearly highlighting the different surface structure dependencies of the two processes.^19^ Notably, grain γ, being close to the {011} family of planes, has the lowest activity in the cathodic window and the highest in the anodic one. Note that the {011} plane is known to possess the strongest oxygen binding within the low-index grains.^51−53^ This characteristic may hinder the ORR but enhance Cu oxidation, possibly by aiding the formation of intermediate oxygenated species.

Examining grain dependence in the presence of BTA-R (grains ε–θ, extracted from Figure 4d,e), the order of activity is ζ > θ > η ≈ ε for the cathodic processes (Figure 5c) and η > θ ≈ ζ > ε for the anodic ones (Figure 5d). This order of activity, as well as the absolute median values of the currents, provides a preliminary indication of the grain-dependent BTA-R action. For the cathodic process, the {001} (α and ε) and {011} (γ and η) grains effectively switch places in the activity ranking when the inhibitor is added, with the {001} being the most active of the pair with no inhibitor (Figure 5a), but having a slightly smaller current density than {011} after the addition of BTA-R (Figure 5c). Quantitatively, the median current is reduced by a factor of 2.7 from α to ε and only by 1.4 from γ to η. Conversely, the activity ranking for the anodic current does not change among the low-index grains, but the activity becomes more uniform, such that the decrease in current density is 1.4 times from α to ε but 2.4 times from γ to η.

The action of BTA-R is evidently both grain and electrochemical-process dependent, with the inhibition effect being stronger on the {001} related planes for the cathodic process and on the {011} for the anodic one. For comparison, {111} related grains (represented by β and ζ) show an intermediate behavior, with the cathodic and anodic current densities decreasing by factors of 1.6 and 1.8, respectively, in the presence of BTA-R. Furthermore, the two selected highly stepped grains included in the distribution plots (δ without inhibitor; θ with BTA-R) are in the same range of current density as the low-index grains, but are distinctive in relative activity. As an example, grain δ is the second most active in both Figure 5a,b, while θ has average activity in both Figure 5c,d.

The observations provided above highlight that the surface-specific behavior of BTA-R on the electrochemistry of Cu cannot be fully elucidated by comparing a few “representative” grains. In fact, even the “low-index” grains compared above differ slightly. For example, grain α has average miller indexes of {0.043 0.010 0.999}, whereas grain ε has average miller indexes of {0.135 0.198 0.971}, which is closer to the {1 2 10} orientation rather than the {001}. In addition, highly stepped grains, which make up a significant part of a polycrystalline surface, present their own unique behavior.

To gain a clearer view of the effective structure-dependency of BTA-R inhibition action necessitates a comprehensive examination of the whole spectrum of crystallographic orientations. Thus, 2D correlation plots of crystallographic orientation (represented by points in the Cartesian plane) and the electrochemical activity (represented by a color scale of the points) were constructed.^19,39^ The 2D projection represents each grain as a point on a scatter plot, with coordinates *C*_1_ and *C*_2_, delimited by three vertexes (representing the low-index grains, {001}, {011}, and {111}) and three sides (representing the {0*n*1}, {*n*11}, and {*nn*1} families of orientations). A more detailed description of the projection, as well as a representation of the key features of the graph, is given in Supporting Information, Section S.5. Specifically, the spatially resolved electrochemical data from Movies S1 and S2 (Figures 4a,b and S8a,b, respectively) were correlated with the corresponding EBSD maps in Figures 4c and S8c to create the plots for the inhibitor-free case, while data from Movies S3 and S4 (Figures 4d,e and S8d,e, respectively) were correlated with the corresponding EBSD maps in Figures 4f and S8f to create the plot with the inhibitor (BTA-R) present. Note that grain-specific data (i.e., Euler angles, Miller indices, *C*_1_, *C*_2_, anodic and cathodic current densities, grain size, and grain orientation spread) are tabulated in theSupporting Information, Section S.6.

### Grain-Dependent BTA-R Action on the Cathodic Process

**Figure 6:**
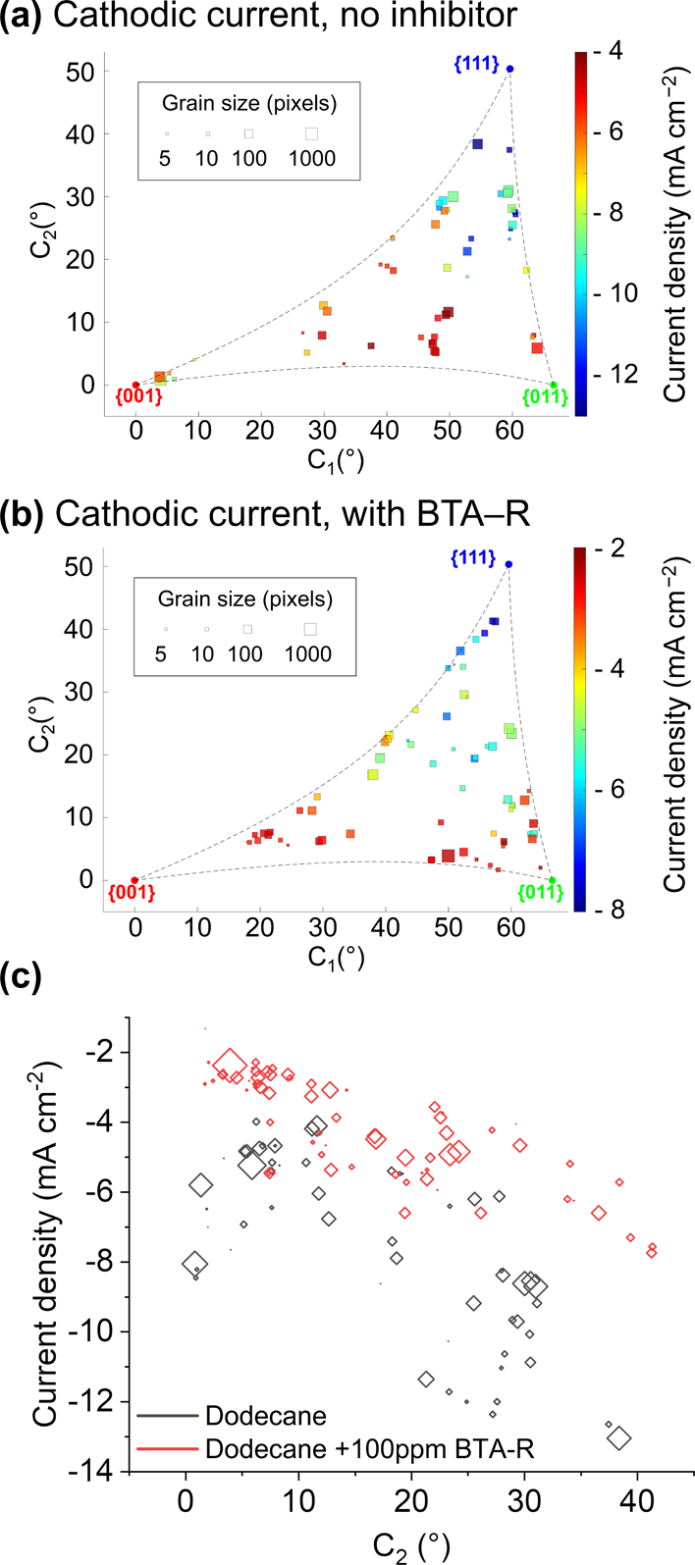
Correlation plots between the crystallographic orientation and the median current density of the cathodic processes (i.e., at −0.44 V) (a) in the absence and (b) in the presence of BTA-R in the dodecane phase. The plot shown in (a) was elaborated from the correlation of Movies S1 and S2 (Figures 4a and S8a) with the IPFz EBSD maps reported in Figures 4c and S8c, while the plot shown in (b) was obtained from the correlation of Movies S3 and S4 (Figures 4d and S8d) with the IPFz EBSD maps reported in Figures 4f and S8f. (c) Correlation plot of the current density at −0.44 V as a function of the sole *C*_2_ coordinate, extracted from both (a) (gray shapes) and (b) (red shapes).

As discussed above, the main reaction involved in the cathodic wave is the ORR, which, in acidic media (such as the pH ≈ 2 employed in this work), follows the 2e^–^ pathway to form H_2_O_2_ as the main product.^54,55^ The 2D cathodic current/crystallographic orientation correlation plots, in the absence and in the presence of BTA-R, are shown in Figure 6a,b, respectively. Without the inhibitor, the grain dependency follows a similar trend to that observed previously under galvanostatic conditions.^19^ The most active grains (i.e., more negative cathodic current density) have high values of *C*_2_ (roughly *C*_2_ ≥ 35°), that is, corresponding to grains that are closer to the {111} orientation; the cathodic activity gradually decreases toward lower values of *C*_2_, that is, toward the {001} and {011} planes. This is consistent with previous macroscopic studies of ORR on Cu{111} and {001} surfaces under similar conditions (but without the triple-phase configuration).^56,57^ However, this study provides further insights: the grains with the smallest cathodic current density magnitude (≈−4 mA cm^–2^; *cf.* ≈13 mA cm^–2^ for the grains closest to {111}) have an orientation between {001} and {011}, located in Figure 6a at 45° < *C*_1_ < 52° and 2° < *C*_2_ < 15°, and centered around two different orientations, {0.161 0.519 0.840} and {0.086 0.505 0.858} [corresponding to (49.38 11.18) and (47.19 6.81), respectively, in the coordinates of the projection]. Interestingly, the two relatively large grains close to the {001} orientation (numbered as grain 15 and 44 in Figure S9; one of them is grain α, Figure 4c) have detectably different activity, with current density on the order of −8 ± 1 and −5.8 ± 0.5 mA cm^–2^. The same pattern is also observed for the smaller grains present in the same region of the orientation spectrum (numbered 16, 19, 31, and 35 in Figure S9).

With the addition of BTA-R to the oil (Figure 6b), grains possessing high values of *C*_2_ still exhibit the most negative (i.e., the largest) current densities with the activity tending to decrease toward the {001} and {011} regions. For the sets of grains examined in this work, there is a decrease in the absolute value of the current density (i.e., a decrease in ORR activity) in the presence of BTA-R, but the relative decrease is not uniformly distributed within the different grains, as already evident from Figure 5.

By plotting the cathodic current density versus the *C*_2_ coordinate alone (Figure 6c), it is evident that, for *C*_2_ ≤ 10° and *C*_2_ ≥ 20°, BTA-R has a very noticeable impact on the cathodic process, with a decrease in current density that is more pronounced the closer the grains are to the {111} orientation. However, in the intermediate band of grains (10° < *C*_2_ < 20°), the BTA-R is not as effective in impeding ORR.

### BTA-R Action on the Anodic Processes

**Figure 7:**
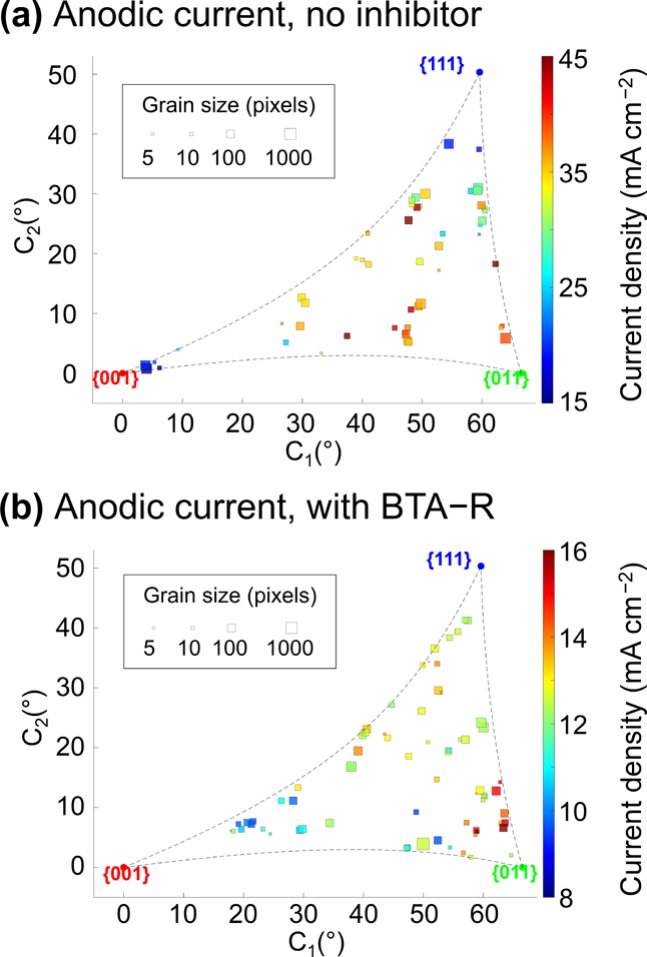
2D correlation plots between the crystallographic orientation and the median current density of the anodic processes (i.e., at +0.24 V) (a) in the absence and (b) in the presence of BTA-R in the dodecane phase. The plot shown in (a) was elaborated from the correlation of Movies S1 and S2 (Figures 4b and S8b) with the IPFz EBSD maps reported in Figures 4c and S8c, while the plot shown in (b) was obtained from the correlation of Movies S3 and S4 (Figures 4e and S8e) with the IPFz EBSD maps reported in Figures 4f and S8f.

As already established above (simple comparisons in Figure 5), the grain dependence of the anodic processes, with and without BTA-R, is dramatically different from the cathodic reactions, further demonstrated by the 2D correlation plots in Figure 7. Without BTA-R (Figure 7a), grains close to the {001} orientation (*C*_1_ < 10°) possess the lowest oxidation susceptibility,^19^ with an average current density of ≈17 mA cm^–2^, followed by those very close to the {111} orientation (*C*_2_ ≥ 35°), with a current of ≈21 mA cm^–2^. For the grains close to the low-index planes, the {011} related grains appear to be the most active (with an average current of ≈37 mA cm^–2^), but the full 2D plot reveals that highly active grains are scattered throughout the entire “high-index” middle region of the plot. Among these, six “high-index” grains show higher activity than for {011} related grains (i.e., current density > 42 mA cm^–2^). Thus, the stepped nature of these structures, together with other factors discussed below, produce increased anodic dissolution characteristics.

While the addition of BTA-R again causes a decrease in the electrochemical activity (corrosion susceptibility), the effect is more pronounced on some surfaces than others (Figure 7b). In the absence (Figure 7a) or presence (Figure 7b) of BTA-R, the grains with the lowest oxidation activity (i.e., the smallest current density) are those of orientation trending toward the {001} plane. Note, however, because the experiments cover different sets of grains (e.g., no grain was collected at a *C*_1_ coordinate < 15° in the presence of BTA-R, with the lowest activity measured over 15° ≤ *C*_1_ ≤ 30°), this conclusion remains cautious. Grains with the highest oxidation current density in the presence of BTA-R are those tending toward the {011} orientation (referring to the grains with *C*_1_ ≥ 60° and *C*_2_ ≤ 15°), which is similar to the case without inhibitor. Different from the case without inhibitor, grains tending toward the {111} orientation (referring, as in the previous case, to grains with *C*_2_ ≥ 35°), present an anodic current density that is roughly halfway between that for the {001} and {011} planes. For example, grains in the {001} and {011} areas experience a *ca.* 50% decrease in current density in the presence of BTA-R, versus a *ca.* 20% decrease for those in the {111} area, indicating less efficient inhibition efficiency on the latter crystallographic planes.

Regarding the “high-index” stepped surfaces, it is interesting to note that these have no “outlier” high activity grains when BTA-R is present (i.e., grains in the middle region of the 2D plot possess activities that are intermediate between the low-index grains). This suggests that BTA-R is very effective on these particular highly stepped surfaces. The action of BTA-R on the spectrum of grains in this middle region of the plot is surface-dependent. For instance, in the grains at relatively high values of *C*_2_ (25° ≤ *C*_2_ ≤ 35°), BTA-R decreases the current density by *ca.* twofold, compared to a *ca.* threefold decrease for grains with *C*_2_ ≤ 20° and 25° ≤ *C*_1_ ≤ 55°. This contrasts with the effect of BTA-R on the cathodic process, where the family of grains exhibiting the lowest inhibition efficiency was 10° < *C*_2_ < 20° and where grains that were closer to the {111} orientation showed the strongest inhibition from BTA-R.

According to previous STM studies of BTA^–^ interaction with Cu single-crystal surfaces, the supramolecular structures formed on the {111} and {011} planes exhibit key differences: on the {011} plane at low coverage, the inhibitor has been observed to lie both parallel to the surface, alongside a perpendicular orientation, which is commonly observed on both planes.^28,29,58^ Linking these observations to the SECCM measurements, suggests that for those surfaces where these different inhibitor-surface structures arise leads to an improvement in the inhibitory effect towards the ORR, but to a diminished effect toward the Cu electro-dissolution.

### Outliers of the Grain-Dependent Action of BTA-R

Crystallographic orientation is not the only factor that influences electrochemical activity; for example, the presence of grain boundaries or slip bands,^59^ crystallographic misorientation,^60^ or physical defects such as microscratches^19^ (although the latter is not so evident for the studies herein, probably due to a relatively low lateral resolution of the SECCM imaging employed to cover large areas of the surface) may also play important roles. As highlighted above, some grains present electrochemical activities that appear to be outliers when compared with other grains of similar orientations. Furthermore, the experimental conditions employed in the EBSD analysis imply a penetration depth of ≈70 nm,^61^ which make the crystallographic measurements relatively immune to the modifications caused by the electrochemical analysis, but does not provide information about the local surface-level orientation modifications (i.e., at the level of the oxide layer). In some cases, such modifications may be more deeply embedded in the structure (*vide infra*), but it is reasonable to consider that because the surface is more similar to an applied material rather than to an ideal atomically flat single-crystal, all grains would show a similar degree of structural non-ideality, reflected in the general activity profiles, that includes contributions from local misorientation.

**Figure 8:**
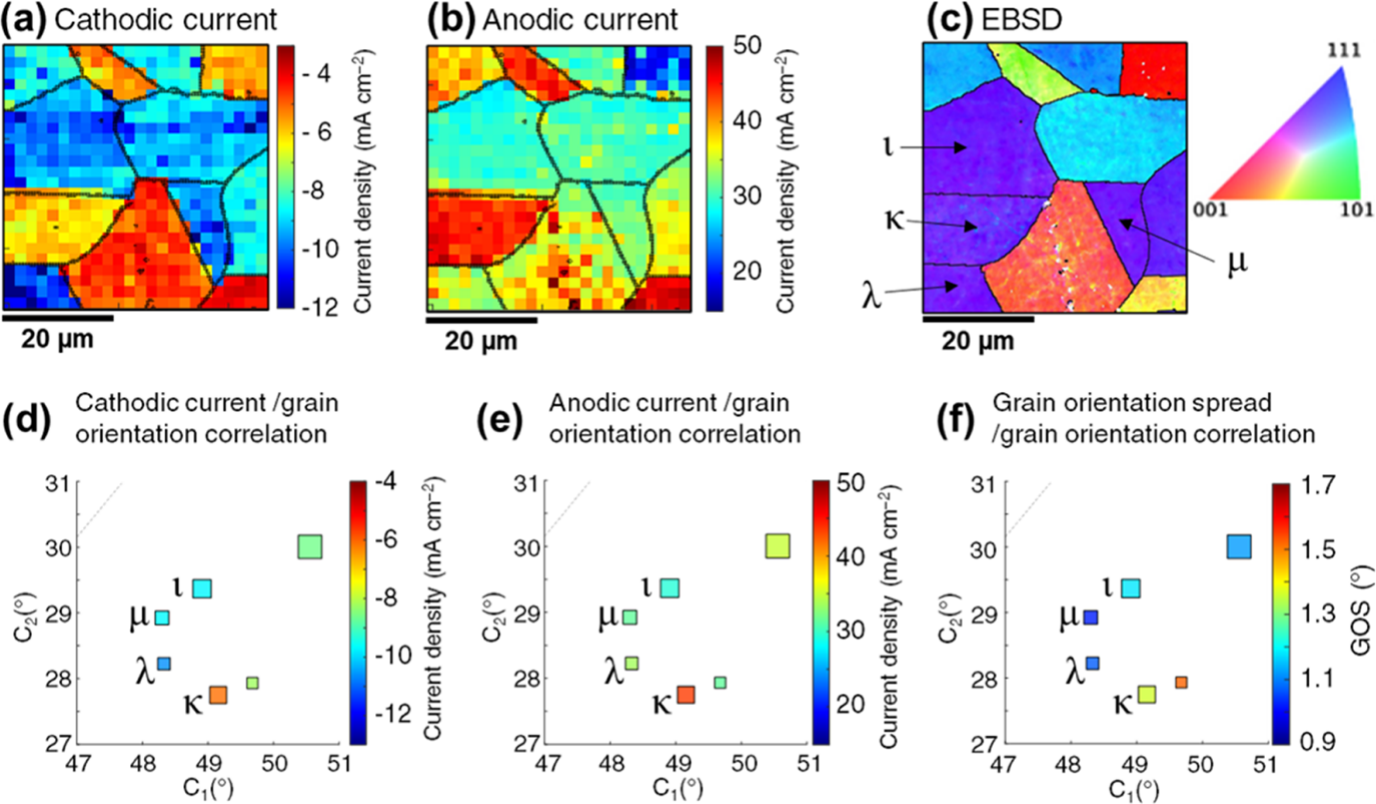
(a,b) Extracts of current density maps from Movie S2, respectively, (a) at −0.44 V (full image in figure) and (b) at +0.24 V (full image in Figure S8b). (c) Corresponding IPFz EBSD map (extract of Figure S8c). (d–f) Extracts of 2D correlation plots shown in (d) Figure 6a and (e) Figure 7a with a magnified scale, with the addition of (f) grain orientation spread of the same grains (obtained from Figures 4c and S8c; the full plot is shown in the Supporting Information, Figure S11).

Figure 8a,b reports single frames of Movie S2, extracted at *E* = – 0.44 V (ORR, Figure 8a) and *E* = +0.24 V (Cu oxidation, Figure 8b), compared to the co-located EBSD map in Figure 8c (the complete images are shown in the Supporting Information, Figure S8a–c). The selected group of grains (marked ι to μ in Figure 8c) present near-identical average crystallographic orientations, as shown by the electrochemistry/structure correlation map in Figure 8d–f (they differ from each other by <2° in the projection’s coordinates), and yet the recorded current density is significantly different on grain κ compared to the other three. Specifically, grain κ presents a less negative/more positive current density in both cases (≈−4 mA cm^–2^ in Figure 8d and ≈+15 mA cm^–2^ in Figure 8e). In other words, grain κ is less active than the others for the ORR, but more active for Cu oxidation. This indicates that the average crystallographic orientation may not be the sole descriptor of ORR and Cu oxidation susceptibility.^62^ Delving further into the EBSD data, grain κ possesses a higher grain orientation spread (i.e., GOS, indicative of the intra-grain standard deviation in crystallographic orientation) than the others, as shown in Figure 8f. A higher GOS implies a higher contribution by planes that are different from the average.

The grains discussed in Figure 8 clearly illustrate that a single grain cannot be confidently taken as exemplar of an entire population of orientations. The anthological comparison of a few selected grains (i.e., low-index planes), with and without inhibitor, is not sufficient for the evaluation of the electrochemical activity of a polycrystalline sample. A comprehensive grain-dependent analysis, which covers as many crystallographic orientations as possible, is necessary to more fully understand trends of corrosion resistance/susceptibility and inhibition efficiency.

## Conclusions

A multi-microscopy screening strategy, involving cross-correlated electrochemical and crystallographic orientation data from SECCM and EBSD, respectively, has been used to elucidate the action of an oil soluble benzotriazole derivative (BTA-R) against the anodic and cathodic processes relevant to Cu corrosion in a triple phase Cu/aqueous nanodroplet/mineral oil system. A key aspect of the experimental configuration is that it closely mimics an automotive/industrial corrosion environment. The Cu surface was covered with a layer of dodecane, with or without 100 ppm of BTA-R dissolved, and the analysis carried out with cyclic voltammetry though an SECCM droplet cell containing H_2_SO_4_ (pH = 2). This approach has allowed the comparison of surface activity toward predominantly ORR on the cathodic side and Cu electro-dissolution on the anodic side. A complex grain-dependent action of BTA-R has been demonstrated, highly dependent on the specific electrochemical process considered. An important aspect of this study is that it was possible to access high-index surface sites that have rarely been studied but are abundant on surface of polycrystalline metals.

In general, BTA-R shows a strong grain-dependent and process-dependent efficacy, both on grains that are relatively close, in orientation, to the low-index grains ({001}, {011}, and {111}) and representative “high-index” grains with stepped surfaces. Interestingly, while BTA-R strongly inhibits the ORR on {111} related grains, it has a much smaller effect on Cu oxidation. Conversely, on selected high-index grains the opposite behavior is observed, with BTA-R strongly inhibiting anodic dissolution, but having less effect on ORR. Comprehensive analysis, for a wide variety of grain orientations, has shown that specific grains may present outstanding (outlier) activity (especially regarding the Cu oxidation reaction) when compared with grains of similar orientation. This could be due to particularly active step edges on the surface, as well as contributions of crystallographic facets that are different from the average of the grain (suggested by a higher grain orientation spread). Crucially, BTA-R appears to intervene most strongly on these grains, leveling the activity to that of similarly oriented grains, and suggesting evidence of step edge action.

Overall, the screening methodology described herein reveals the surfaces and reactions of the corrosion process that are more sensitive to inhibition to BTA-R. Further studies, in close association with surface science and modeling, may lead to a deeper understanding of what kind of interactions between an inhibitor and surface are the most effective in impeding electrochemically driven corrosion processes, and eventually enable the design and development of new and more efficient corrosion inhibitors.

## Supporting Information Available

Electrochemical movie for the system without BTA-R, Movie S1 (AVI)
Electrochemical movie for the system without BTA-R, Movie S2 (AVI)
Electrochemical movie for the system with BTA-R, Movie S3 (AVI)
Electrochemical movie for the system with BTA-R, Movie S4 (AVI)
Additional experimental details, oxide layer reduction calculations, additional voltammetric comparisons, effect of BTA-R on surface wetting, movies’ captions, grain orientation-2D projection, and additional maps and grain lists (PDF)

## Supplementary Material

jp2c04494_si_001.avi

jp2c04494_si_002.avi

jp2c04494_si_003.avi

jp2c04494_si_004.avi
