# Supplementary material for: Screening the Surface Structure-Dependent Action of a Benzotriazole Derivative on Copper Electrochemistry in a Triple-Phase Nanoscale Environment
Source: J Phys Chem C Nanomater Interfaces. 2022 Aug 29;126(35):14897–907. doi: 10.1021/acs.jpcc.2c04494 (PMC9465680; doi:10.1021/acs.jpcc.2c04494)
Supplement: Supplementary file 5 — jp2c04494_si_005.pdf [file jp2c04494_si_005.pdf]

Supporting information for

Screening the Surface Structure-Dependent Action of a Benzotriazole Derivative on Copper Electrochemistry in a Triple-Phase Nanoscale Environment

Enrico Daviddi,[†] *Viacheslav Shkirskiy,*[‡] *Paul M. Kirkman,*[§] *Mathew P. Robin,*[§] *Cameron L. Bentley*[*,||] *and Patrick R. Unwin*[*,†]

[†]Department of Chemistry, University of Warwick, Coventry CV4 7AL, U.K.
[‡]Université Paris Cité, ITODYS, CNRS, F-75006, Paris, France
[§]Lubrizol LTD, Nether Ln, Hazelwood DE56 4AN, UK
[||]School of Chemistry, Monash University, Clayton, Victoria 3800, Australia
*E-mail: cameron.bentley@monash.edu (C.L.B.); p.r.unwin@warwick.ac.uk (P.R.U.)

Contents

S.1 Additional Experimental Details S3

S.2 Oxide layer reduction calculations S4

S.3 Additional Voltammetric Comparisons S7

S.3 Effect of BTA–R on surface wetting S8

S.4 Movies Captions S10

S.5 Grain orientation-2D projection S11

S.6 Additional maps and grain lists S13

References S18

S.1 ADDITIONAL EXPERIMENTAL DETAILS

Pipette pulling parameters. All pipettes were pulled to a size of approximately 400 nm diameter with the following pulling parameters:

line 1: HEAT 340, FIL 3, VEL 30, DEL 250, PUL -;

line 2: HEAT 340, FIL 3, VEL 30, DEL 210, PUL 120.

Macroscale electrochemistry. Macroscopic linear sweep voltammetry was performed in a conventional three-electrode mode, employing a 3 mm diameter Cu macrodisk electrode (area 0.071 cm^2, Bioanalytical System, U.S.A.) as a working electrode, a coiled platinum wire (0.5 mm diameter, Goodfellow, U.K.) as counter electrode and a commercial Ag/AgCl electrode (see above) as reference electrode. In the case of the deaerated experiment, the solution was purged with Ar prior to experimentation, and a blanket of Ar was maintained during the course of the voltammetry. Experiments were performed with a commercial CHI400 (CH Instruments, U.S.A.) potentiostat.

Contact Angle measurements. Contact angle measurements were carried out with Krüss DSA30 goniometer and software. The Cu sample was placed in a quartz cuvette filled with the mineral oil solution (either dodecane or 100 ppm BTA−R in dodecane), and the droplet was placed on it subsequently. Droplets had a volume of 300 μL and were mechanically laid on the Cu surface. The contact angle was monitored every 0.5 s for 100 s and reported as the average between the left and the right angle.

S.2 OXIDE LAYER REDUCTION CALCULATIONS

Polished Cu surfaces develop an oxide layer upon exposure to air, with a thickness and composition that depends on the exposure time and atmospheric conditions. A thin layer of Cu(I) oxide, Cu_2O (of approximately 2.5 nm)[1-2] forms immediately after polishing, and a further layer of Cu(II) oxide, CuO, grows on the top of it with slower kinetics (i.e., thickness of ≈5 nm after 2 months exposure).[3] Under the present conditions (the sample being analyzed one day after the polishing procedure and stored under ambient conditions), a native oxide layer (denoted CuO_x, herein) of approximately 2-3 nm can reasonably be assumed. Assuming an oxide layer thickness of 3 nm and taking density and molar mass values of 6.0 g cm^{-3} and 143.09 g mol^{-1}, respectively for Cu_2O and; 6.31 g cm^{-3} and 79.545 g mol^{-1}, respectively for CuO; charge densities of ≈ 2.5 mC cm^{-2} and ≈ 4.6 mC cm^{-2} can be calculated for Cu_2O and CuO, respectively. Thus, 2.5 mC cm^{-2} is taken to be the lower limit of charge required to remove a mixed-valence CuO_x film of thickness 3 nm from Cu. It is further possible to estimate the number of "layers" of oxide considering that Cu_2O has a cubic crystallographic structure, with a lattice constant $a \approx 4.26$ Å, with each cell containing effectively 4 Cu atoms, arranged in an fcc sublattice. Therefore, taking the (001) surface as a model, there will be a layer containing two coplanar Cu^+ ions every half of the lattice constant, *i.e.* ≈ 2.13 Å. Assuming that a layer of Cu_2O is coincident with a layer of Cu^+ ions, in a 3 nm oxide layer, there will be approximately 14 Cu_2O monolayers, corresponding to a charge density of ≈ 0.2 mC cm^{-2} per monolayer.

In the present study, SECCM meniscus contact was made at − 0.45 V vs. Ag/AgCl/3.4 M KCl (Ag/AgCl hereafter) and held for a period of 0.25 sec before linearly scanning the potential anodically, with the substrate (working electrode) current measured throughout. Median *i*−*t* curves, obtained during the initial landing period, with and without BTA−R, are shown in

Figure S3. Initially (t < 5 ms), both curves present a sharp near-exponential decay, followed by a "shoulder" before entering a region where $i \propto t^{-1/2}$, roughly after 0.1s.

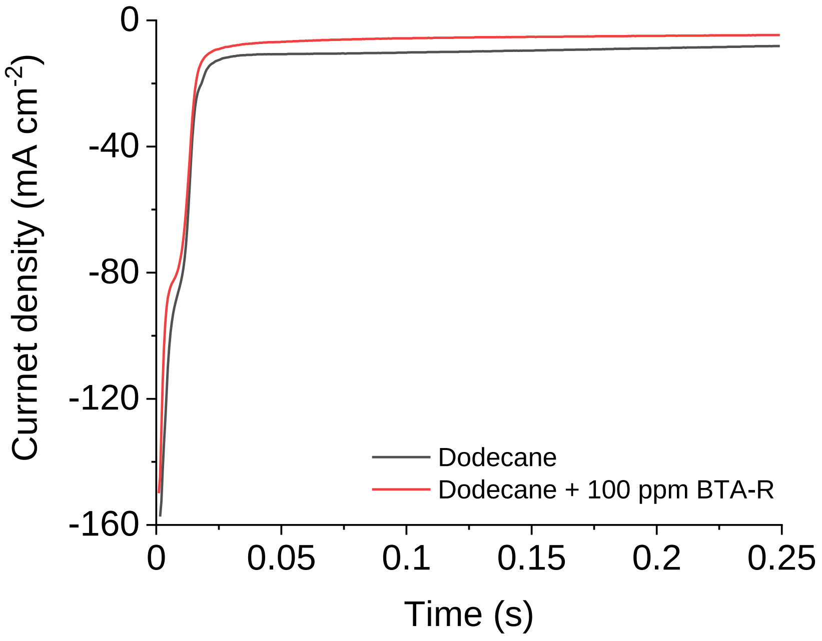

Figure S1. Current-time behavior upon meniscus landing at $E = -0.45$ V for a duration of 0.25 s, both in absence (gray line) and in presence (red line) of BTA−R in the mineral oil phase.

Assigning the first exponential region to non-faradaic processes (e.g., double layer charging and stray capacitance from the measurement set up) and the final region to the cathodic reactions relevant to corrosion (discussed in the main text), the "shoulder" is assigned to the (partial) reduction of the native oxide layer. Integrating over this region produces median values of 0.63 and 0.62 mC cm^{-2} without and with BTA−R, respectively, which corresponds to approximately 3-4 monolayers of Cu_2O (*vide supra*). This is a fraction of the charge that would be required to fully reduce the native oxide layer (2.5 to 4.6 mC cm^{-2}, assuming 3 nm thick layers of Cu_2O or CuO, respectively).[4] This analysis indicates that the native oxide layer is converted into a complex, mixed layered structure, consisting of an electro-reduced Cu(0) overlayer that protects the underlying CuO_x from further reduction, giving rise to a $Cu_o|CuO_x|Cu_b$ sandwich structure ("o" and "b" refer to overlayer and bulk Cu, respectively), similar to what has previously been proposed by Nakayama *et al.*[5]

Interestingly, the calculated oxide reduction charge appears to be grain dependent, as shown by the maps in Figure S2, respectively for the case without (Figure S2a and b) and with (Figure S2c and d) BTA-R. However, as shown by the point by point correlation plots there is only a very weak correlation between the landing pulse charge and the following Cu electro-oxidation current ((Figure S2e and f, respectively, for the case without and with BTA−R), especially when the inhibitor is added.

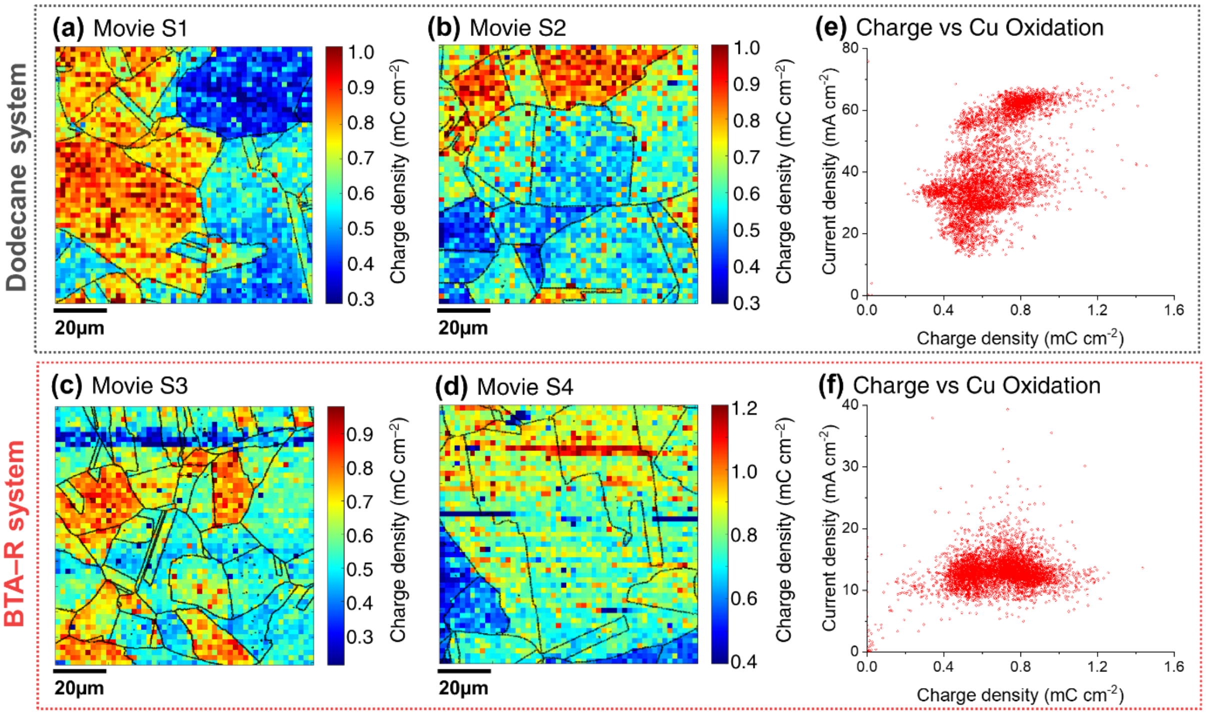

Figure S2. **(a-d)** Electrochemical images extracted from Movies S1 - S4, showing the total oxide reduction charge passed during the 0.25 s landing period at – 0.45 V, respectively for **(a-b)** the measurement without BTA−R and **(c-d)** the measurement with BTA−R. All SECCM measurements were conducted with a 10 mM H_2SO_4 solution in the probe. For clarity, the grain boundaries of the corresponding crystallographic orientation map were overlapped to each electrochemical image, extracted, respectively, from (a) Figure 4c, (b) Figure S8c, (c) Figure 4f and (d) Figure S8f. **(e)** Point by point correlation between the charge extracted from (a) and (b) with the corresponding anodic current extracted from Figure 4b and Figure S8b, for the case without BTA−R. $R^2 = 0.297$. **(f)** Point by point correlation between the charge extracted from (c) and (d) with the corresponding anodic current extracted from Figure 4e and Figure S8e, for the case with BTA−R present. $R^2 = 0.019$.

S.3 ADDITIONAL VOLTAMMETRIC COMPARISONS

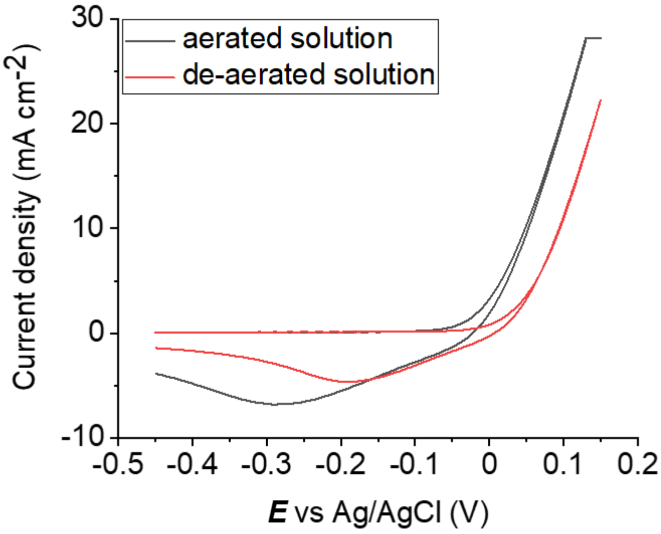

Figure S3. Macroscopic CVs obtained from a 3 mm diameter Cu macrodisk electrode in aerated (black curve) and deaerated (red curve) 1 M H_2SO_4. The potential was swept from − 0.45 V to + 0.15 V vs Ag/AgCl, at a sweep rate of 1 V s^{-1}. In both cases, a potential pulse of 60s was applied at the initial potential prior to the start of the sweep. The two curves present a difference in the onset potential for Cu electro-dissolution of ≈ 50 mV.

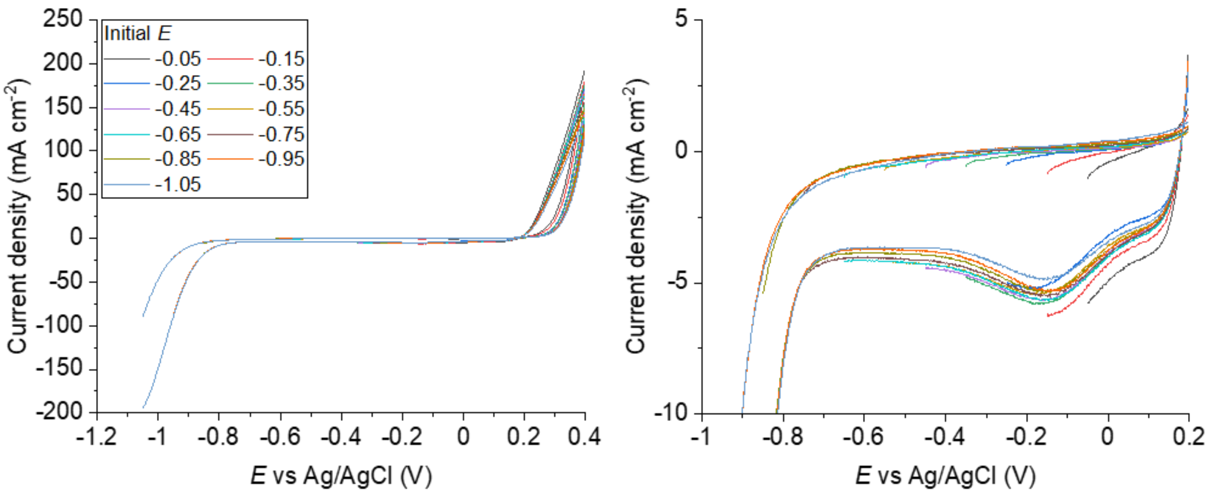

Figure S4. CVs obtained within the SECCM triple-phase setup described in the main text, at a sweep rate υ = 1 V s^{-1}, on a polycrystalline Cu surface immediately after a landing period of 0.25 s at different initial cathodic potentials (marked on the figure), in de-aerated conditions. The presented curves are the median values from a SECCM experiment, with N = 102 in each case. These measurements were obtained in the SECCM configuration with a nanopipet probe containing 0.01 M H_2SO_4. Note that the E scale refers to Ag/AgCl (3.4 M KCl). The plots on the right are a zoomed version of those on the left.

S.3 EFFECT OF BTA–R ON SURFACE WETTING

Effect of the corrosion inhibitor on surface wetting. According to complementary SEM imaging of the scan areas (shown in FigureS4a and b respectively in absence and presence of BTA−R), the contact area of the droplet decreases when the inhibitor is added. In particular, the average of 10 randomly selected droplet footprints reveals a contact area of, respectively, 3.79×10^{-9} cm^2 in the un-inhibited case, corresponding to a diameter of 695 nm, and 3.47×10^{-9} cm^2 in the inhibited case, corresponding to a diameter of 665 nm (note that the same probe was used in the two experiments). This is compatible with BTA−R forming a uniform layer on the surface, exposing the aqueous droplet to a layer of organic molecules; such a layer increases the hydrophobicity, decreasing the extent of the nanodroplet spread during contact with the surface.

This conclusion can also be supported, to some extent, by contact angle measures, as shown in Figure S5. The contact angle measurement was conducted in a triple-phase environment similar to that used for the SECCM measures, with a 10 mM H_2SO_4 solution droplet immersed in mineral oil and in contact with a Cu surface polished to a mirror finish. The contact angle increased about ≈ 20° when the BTA−R was added in the dodecane, with a considerable decrease in the interfacial tension between the droplet and the metal. This behavior is compatible with the formation of a hydrophobic coating. However, such effect does not have a qualitatively appreciable dependency on grain orientation, as shown by the SEM images of droplet footprints (Figure S4). To aid comparison of behavior with and without BRA-R, current density are used based on the observed footprint sizes.

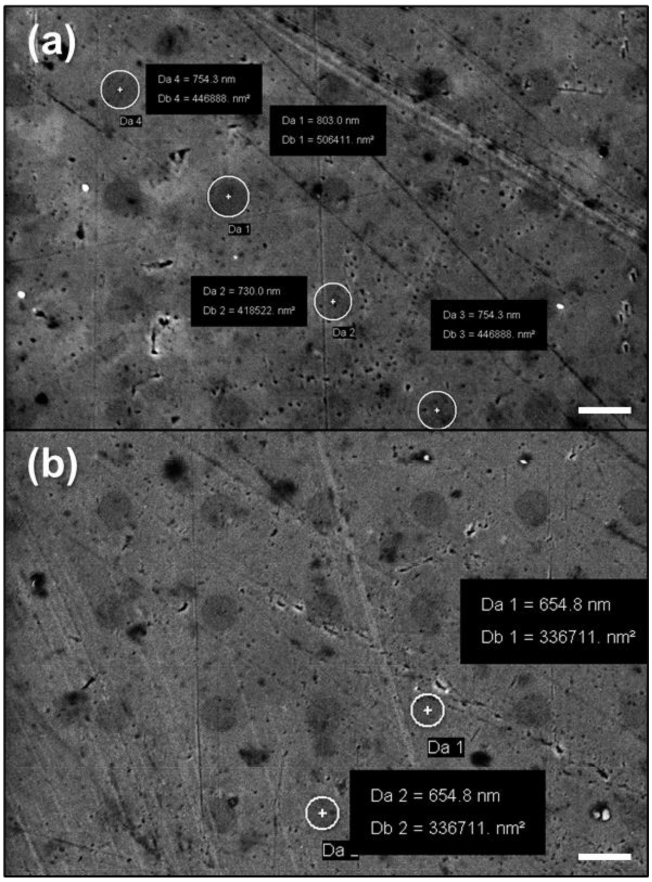

Figure S5. Droplet footprints recorded with SEM after SECCM scans either in the absence (a) or presence (b) of BTA–R in the oil layer. Some selected droplet footprints are marked with a circle in both figures, with their sizes reported aside. The scale bar in the SEM images corresponds to 1 µm.

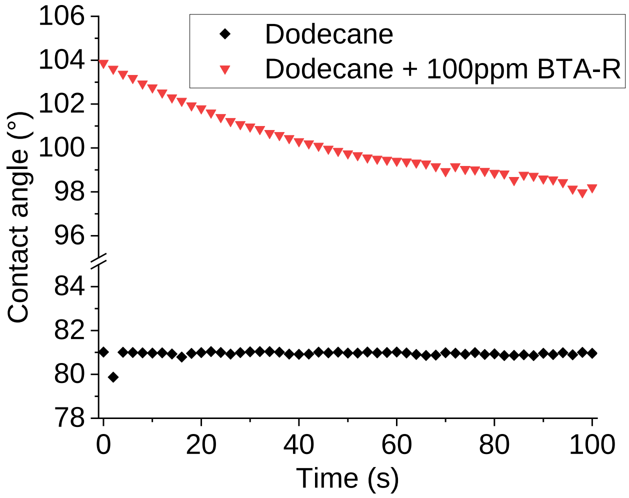

Figure S6. Contact angle measurements obtained with a 10 mM H_2SO_4 solution droplet (300 µL) deposited on a polycrystalline Cu surface (mirror-finish) covered in pure dodecane (black spots) or a 100 ppm solution of BTA−R in dodecane (red spots). The contact angle was measured every 2 second and it is an average between the left and the right side of the droplet.

S.4 Movies Captions

Movie S1. Electrochemical movie, recorded in the SECCM configuration (51 by 51 pixels, hopping distance 2 μm), with pure dodecane acting as the mineral oil layer, showing electrochemical activity across a polycrystalline Cu surface. Each pixel represents a single CV, obtained with E_i = – 0.440 V vs. Ag/AgCl, E_f = 0.255 V and υ = 1 V s^{-1}, collected after a landing period of 0.25 s at E_i. The movie is shown with a potential resolution of 0.01 V per frame. Each frame at a given potential, E_x, is obtained by averaging the current between E_x − 0.005 V and E_x + 0.005 V. The nanopipet probe used for SECCM contained aerated 0.01 M H_2SO_4, was ≈ 400 nm in diameter and contacted an average area of 3.79 × 10^{-9} cm^2. The grain boundaries extracted from the EBSD map in Figure 4c of the main text are overlapped onto each frame of the movie.

Movie S2. Electrochemical movie, recorded in the SECCM configuration (51 by 51 pixels, hopping distance 2 μm), with pure dodecane acting as the mineral oil layer, showing electrochemical activity across a polycrystalline Cu surface. Each pixel represents a single CV, obtained with E_i = – 0.455 V vs. Ag/AgCl, E_f = 0.240 V and υ = 1 V s^{-1}, collected after a landing period of 0.25 s at E_i. The movie is shown with a potential resolution of 0.01 V per frame. Each frame at a given potential, E_x, is obtained by averaging the current between E_x − 0.005 V and E_x + 0.005 V. The nanopipet probe used for SECCM contained aerated 0.01 M H_2SO_4, was ≈ 400 nm in diameter and contacted an average area of 3.79 × 10^{-9} cm^2. The grain boundaries extracted from the EBSD map in Figure S7c are overlapped onto each frame of the movie.

Movie S3. Electrochemical movie, recorded in the SECCM configuration (51 by 51 pixels, hopping distance 2 μm), with a 100 ppm BTA−R solution in dodecane acting as the mineral oil layer, showing electrochemical activity across a polycrystalline Cu surface. Each pixel represents a single CV, obtained with E_i = – 0.455 V vs. Ag/AgCl, E_f = 0.240 V and υ = 1 V s^{-1}, collected after a landing period of 0.25 s at E_i. The movie is shown with a potential resolution of 0.01 V per frame. Each frame at a given potential, E_x, is obtained by averaging the current between E_x − 0.005 V and E_x + 0.005 V. The nanopipet probe used for SECCM contained aerated 0.01 M H_2SO_4, was ≈ 400 nm in diameter and contacted an average area of 3.47 × 10^{-9} cm^2. The grain boundaries extracted from the EBSD map in Figure 4f are overlapped onto each frame of the movie.

Movie S4. Electrochemical movie, recorded in the SECCM configuration (51 by 51 pixels, hopping distance 2 μm), with a 100 ppm BTA−R solution in dodecane acting as the mineral oil layer, showing electrochemical activity across a polycrystalline Cu surface. Each pixel represents a single CV, obtained with E_i = – 0.455 V vs. Ag/AgCl, E_f = 0.240 V and υ = 1 V s^{-1}, collected after a landing period of 0.25 s at E_i. The movie is shown with a potential resolution of 0.01 V per frame. Each frame at a given potential, E_x, is obtained by averaging the current between E_x − 0.005 V and E_x + 0.005 V. The nanopipet probe used for SECCM contained aerated 0.01 M H_2SO_4, was ≈ 400 nm in diameter and contacted an average area of 3.47 × 10^{-9} cm^2. The grain boundaries extracted from the EBSD map in Figure S7f are overlapped onto each frame of the movie.

S.5 GRAIN ORIENTATION-2D PROJECTION

The development of the grain orientation 2D projection employed in this work has been previously described in detail.[6] Here a brief recall is given for clarity of understanding. The average orientation of each crystallographic grain present in the scanned areas (measured through EBSD, detailed in section S.1) was extracted as Euler angle data, φ_1, Φ and φ_2. The average miller indexes (*h,k,l*) for each plane were calculated from Φ and φ_2:

$$h = n \sin\Phi \sin\varphi_2 \tag{S1}$$

$$k = n \sin\Phi \cos\varphi_2 \tag{S2}$$

$$l = n \cos\Phi \tag{S3}$$

The miller indexes for each grain were sorted from smallest to largest, for symmetry reasons. (h',k',l', such as $h' \leq k' \leq l'$) and were used to calculate the angles between any generic plane α and each of the three low-index planes employed for the cubic system representation, (001), (011), (111), respectively as γ_1, γ_2 and γ_3:

$$\cos\gamma = \frac{h_1' h_2' + k_1' k_2' + l_1' l_2'}{\sqrt{(h_1')^2 + (k_1')^2 + (l_1')^2}\sqrt{(h_2')^2 + (k_2')^2 + (l_2')^2}} \tag{S4}$$

Therefore, the orientation α could be expressed with a point P of coordinates (γ_1^α, γ_2^α, γ_3^α). Such coordinates, for the three aforementioned low index grains were: for (001), $P_1 = (0,a,b)$; for (011), $P_2 = (a,0,c)$; for (111), $P_3 = (b,c,0)$; with $a = 45^\circ$, $b = 54.736^\circ$ and $c = 35.264^\circ$. Due to the degeneration of the miller indexes (*i.e.*, they are not totally independent), all of the points P calculated for the generic planes α, lay on a hyperbolic plane that passes through P_1, P_2 and P_3. Therefore, as a 2D representation (with coordinates C_1 and C_2) of the orientation of α, the projection of P on the plane passing through P_1, P_2 and P_3 was chosen, in a way that the three low index grains have the following coordinates: P_1 (0,0), representing the {001}//ND plane, P_2 (66.5517°,0), representing the {011}//ND plane, and P_3 (59.6074°,50.336°), representing the

{111} plane. The coordinates C_1 and C_2 of some representative grains relevant for this work, as well as their model 3D representations, are shown in Figure S6.

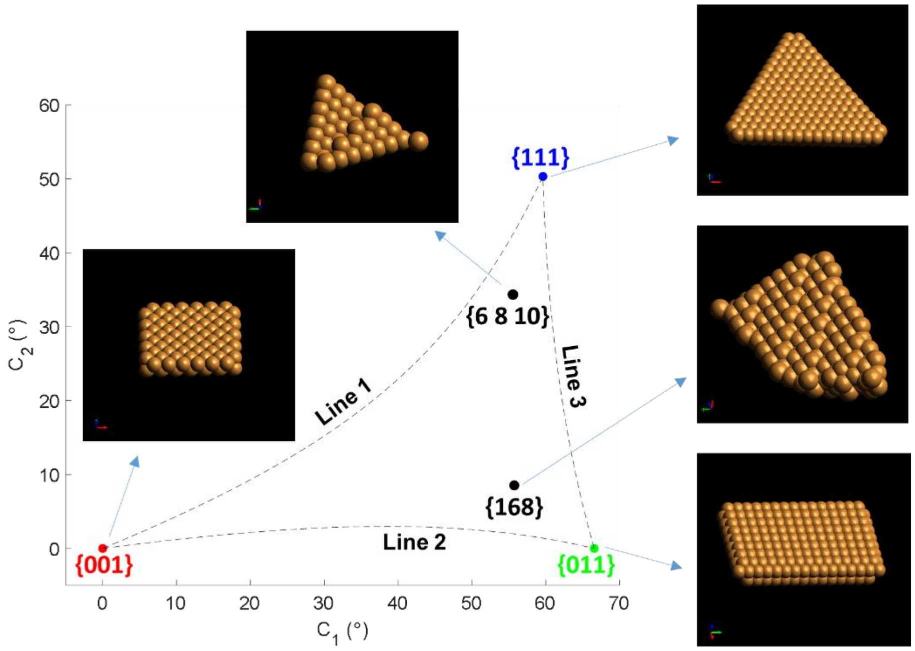

Figure S7. Indication and representation of some relevant grains on the developed 2D orientation projection. In the graph, line 1 represents the family of planes with miller indices (m,m,1), where $0 < m < 1$; line 2 represents the family of planes with miller indices (0,n,1), where $0 < n < 1$; line 3 represents the family of planes with miller indices (q,1,1), where $0 < q < 1$. All grains lay between these three lines.

S.6 Additional maps and grain lists

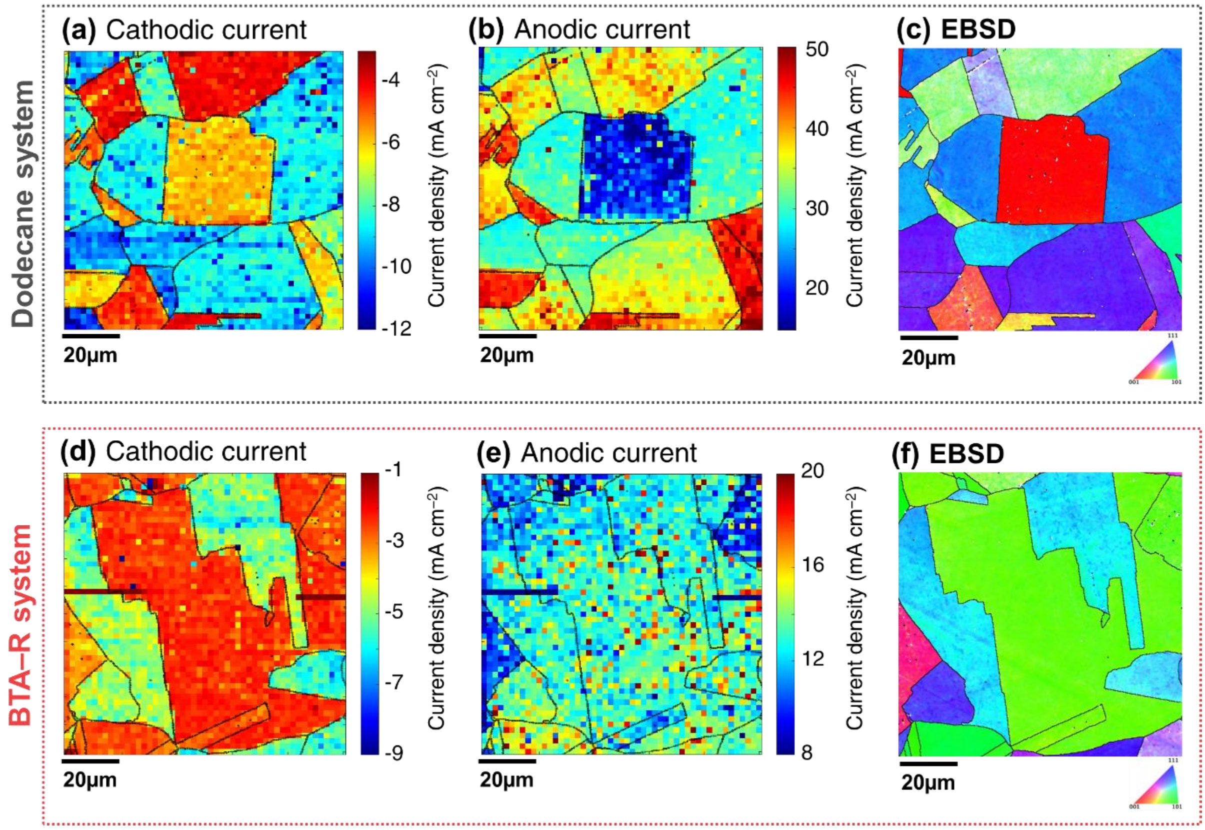

Figure S8. Electrochemical images extracted from Movies S2 and S4, alongside relative crystallographic orientation maps. **(a-b)** Single frames of Movie S1 showing the current density recorded at (a) $E = -\ 0.44$ V and (b) $E = +\ 0.24$ V with pure dodecane as the oil phase **(c)** Co-located crystallographic orientation map, obtained with EBSD after Movie S2 was recorded. **(d-e)** Single frames of Movie S4 showing the current density recorded at (d) $E = -\ 0.44$ V and (e) $E = +\ 0.24$ V with a solution of BTA−R 100 ppm in dodecane as the oil phase. **(f)** Co-located crystallographic orientation map, obtained with EBSD after Movie S4 was recorded. All electrochemical measurements were conducted with a 10 mM H_2SO_4 solution in the probe. For clarity, the grain boundaries of the corresponding crystallographic orientation map were overlapped to each electrochemical image.

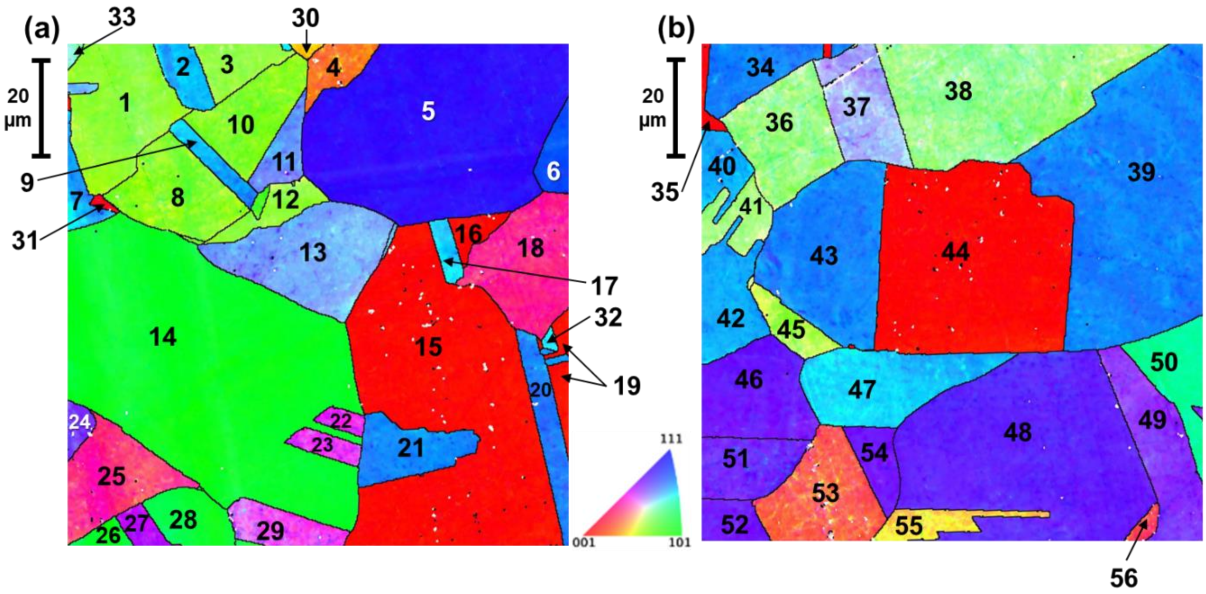

Figure S9. Definition of the grain ID for each grain analyzed from **(a)** Movie S1 (*i.e.*, reproduction of the EBSD data shown in the main text, Figure 4c) and **(b)** Movie S2 *i.e.*, reproduction of the EBSD data shown in the Supporting information, Figure S7c).

Table S1. List of all grains analyzed by SECCM in absence of BTA−R (Movie S1 and Movie S2), with the average Euler angles, Miller indices and Projection Coordinates, cathodic and anodic currents (i_c and i_a, measured at – 0.44 and + 0.24 V, respectively) and the grain orientation spread (GOS) listed for each one. The grain IDs correspond to those defined in Figure S8.

ID	Average Euler angles (˚)			Average Miller indices			Projection Coordinates (˚)		Median i_c (mA cm^{-2})	Median i_a (mA cm^{-2})	Size (px)	GOS (°)
	φ_1	Φ	φ_2	*h*	*k*	*l*	C_1	C_2				
1	320.73	30.87	9.06	0.081	0.507	0.858	47.29	6.53	-4.75	38.01	108	1.78
2	237.96	47.28	27.69	0.341	0.651	0.678	60.40	27.56	-12.00	30.76	26	1.41
3	319.04	31.13	11.34	0.102	0.507	0.856	47.58	7.65	-5.15	37.28	31	1.27
4	211.30	17.45	77.40	0.293	0.065	0.954	27.27	5.14	-6.93	24.88	28	1.76
5	301.28	46.05	46.92	0.526	0.492	0.694	54.45	38.36	-13.04	19.57	368	1.1
6	8.15	49.83	36.11	0.450	0.617	0.645	59.51	37.44	-12.64	19.29	24	1.17
7	238.06	47.26	27.29	0.337	0.653	0.679	60.50	27.17	-12.36	31.04	26	1.95
8	105.62	31.07	83.41	0.513	0.059	0.857	47.57	5.42	-4.83	36.23	86	2.21
9	192.84	47.01	61.68	0.644	0.347	0.682	60.06	27.94	-11.03	29.87	15	1.69
10	105.32	31.23	83.88	0.516	0.055	0.855	47.79	5.21	-4.83	35.43	73	1.66
11	7.49	39.34	30.21	0.319	0.548	0.773	53.43	23.31	-11.72	24.92	24	1.48
12	319.76	30.81	9.68	0.086	0.505	0.859	47.19	6.81	-4.67	38.89	27	1.64
13	60.65	38.18	61.44	0.543	0.296	0.786	52.80	21.28	-11.35	35.59	130	1.28
14	243.74	43.89	6.53	0.079	0.689	0.721	63.94	5.87	-5.23	38.97	571	1.73
15	165.27	2.53	77.12	0.043	0.010	0.999	4.00	0.81	-8.05	15.22	434	1.58
16	169.00	2.78	73.30	0.046	0.014	0.999	4.39	0.99	-8.21	19.08	15	1.93
17	359.38	45.53	26.16	0.315	0.641	0.701	59.72	24.87	-12.00	26.09	13	1.48
18	345.04	20.94	37.88	0.219	0.282	0.934	29.89	12.66	-6.76	34.46	101	1.64
19	156.08	3.93	86.26	0.068	0.004	0.998	6.16	0.90	-8.45	16.75	17	1.26
20	39.24	45.89	57.79	0.608	0.383	0.696	58.18	30.46	-10.07	24.88	37	1.29
21	265.66	47.17	31.01	0.378	0.629	0.680	59.39	30.51	-10.87	28.59	56	1.14

22	152.94	28.66	49.99	0.367	0.308	0.877	38.98	19.19	-5.48	33.82	8	1.79
23	150.96	29.12	51.79	0.382	0.301	0.874	40.03	18.94	-5.48	34.46	16	1.64
24	130.13	38.52	39.08	0.393	0.483	0.782	49.68	27.93	-8.25	31.53	16	1.52
25	349.18	20.91	34.23	0.201	0.295	0.934	30.49	11.77	-6.04	34.62	88	2.46
26	36.02	43.93	81.27	0.686	0.105	0.720	63.39	7.92	-4.67	39.22	11	1.97
27	118.86	31.68	45.59	0.375	0.368	0.851	40.91	23.37	-6.40	35.91	16	1.91
28	35.60	43.60	81.49	0.682	0.102	0.724	63.18	7.61	-6.44	35.59	15	2.17
29	149.77	29.39	54.31	0.399	0.286	0.871	41.04	18.21	-5.39	34.75	36	2.04
30	305.88	21.53	1.97	0.013	0.367	0.930	33.16	3.38	-5.03	30.96	4	0.93
31	78.20	6.48	44.75	0.079	0.080	0.994	9.25	3.99	-7.65	25.20	4	1.52
32	359.56	44.75	24.96	0.297	0.638	0.710	59.52	23.24	-10.27	23.15	4	2.46
33	279.01	36.94	23.85	0.243	0.550	0.799	52.81	17.24	-8.62	35.67	5	1.79
34	23.78	47.27	58.42	0.626	0.385	0.679	59.29	31.10	-9.18	28.83	45	1.26
35	82.32	3.53	54.69	0.050	0.036	0.998	5.33	1.86	-6.48	21.02	8	1.55
36	239.02	32.90	17.19	0.161	0.519	0.840	49.38	11.18	-4.19	36.76	112	1.62
37	344.96	35.01	61.96	0.506	0.270	0.819	49.60	18.67	-7.89	33.02	77	1.33
38	238.52	33.29	17.73	0.167	0.523	0.836	49.80	11.63	-4.11	36.07	234	1.57
39	23.85	47.30	58.59	0.627	0.383	0.678	59.35	30.98	-8.70	29.15	365	1.11
40	252.47	46.88	28.58	0.349	0.641	0.684	59.90	28.08	-8.37	36.88	98	1
41	35.72	31.91	73.17	0.506	0.153	0.849	48.13	10.66	-5.15	43.36	36	1.26
42	104.18	3.82	33.45	0.037	0.056	0.998	5.80	1.96	-7.01	24.32	1	1.61
43	23.83	47.01	58.82	0.626	0.379	0.682	59.24	30.53	-8.54	29.55	178	1.16
44	83.74	2.51	53.53	0.035	0.026	0.999	3.77	1.34	-5.80	19.61	356	1.77
45	302.09	29.63	78.25	0.484	0.101	0.869	45.43	7.58	-5.39	42.64	28	1.93
46	4.87	38.72	41.60	0.415	0.468	0.780	48.91	29.37	-9.70	29.39	90	1.18
47	290.62	46.08	63.60	0.645	0.320	0.694	60.01	25.49	-9.18	29.71	114	1.1
48	299.25	39.95	40.24	0.415	0.490	0.767	50.56	30.00	-8.62	34.95	362	1.12
49	109.53	36.35	51.36	0.463	0.370	0.805	47.74	25.57	-6.20	45.09	90	1.36
50	192.16	46.21	18.58	0.230	0.684	0.692	62.27	18.28	-7.41	44.85	45	1.07
51	177.59	38.13	50.50	0.476	0.393	0.787	49.16	27.75	-6.12	43.56	65	1.36
52	5.70	37.87	41.05	0.403	0.463	0.789	48.33	28.23	-10.63	33.66	24	1.07
53	237.76	19.29	22.03	0.124	0.306	0.944	29.66	7.90	-4.67	35.99	101	2.14
54	4.69	38.18	41.88	0.413	0.460	0.786	48.30	28.92	-9.66	31.49	33	1.02
55	49.55	24.19	79.12	0.402	0.077	0.912	37.48	6.24	-3.99	45.21	30	1.54
56	164.91	17.53	27.44	0.139	0.267	0.954	26.60	8.30	-5.23	37.12	6	2.12

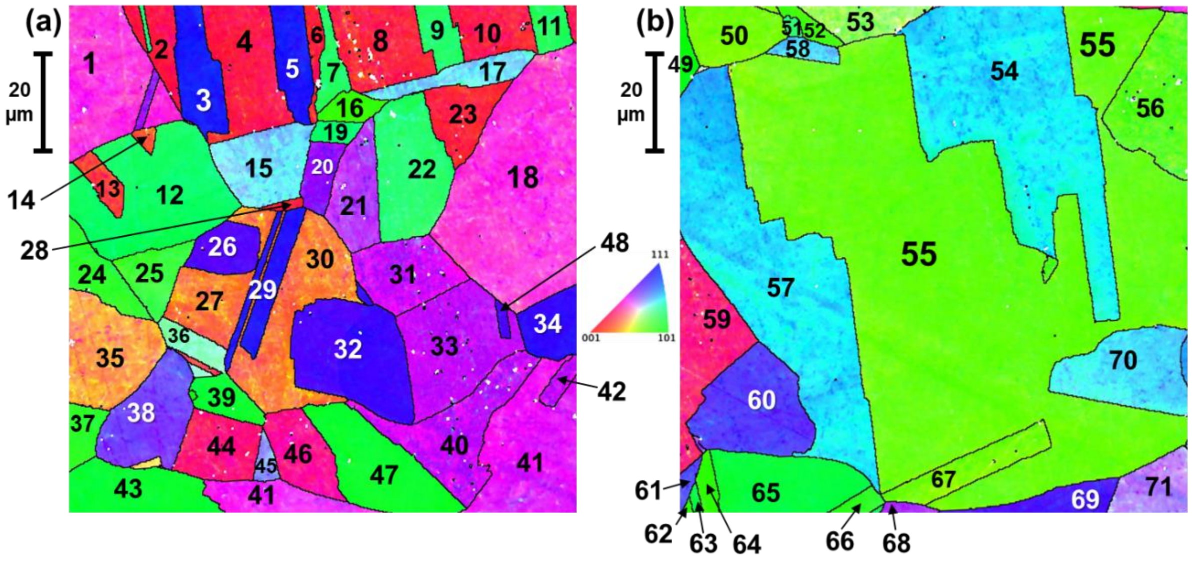

Figure S10. Definition of the grain ID for each grain analyzed from **(a)** Movie S3 (reproduction of Figure 4f) and **(b)** Movie S4 (reproduction of Figure S7f).

Table S2. List of all grains analyzed by SECCM in presence of BTA−R in the mineral oil phase (Movie S3 and Movie S4), with the average Euler angles, Miller indices and Projection Coordinates, cathodic and anodic currents (measured at – 0.44 and + 0.24 V, respectively) and the grain orientation spread (GOS) listed for each one. The grain IDs correspond to those defined in Figure S9.

ID	Average Euler angles (°)			Average Miller indices			Projection Coordinates (°)		Median i_c (mA cm^{-2})	Median i_a (mA cm^{-2})	Size (px)	GOS (°)
	φ_1	Φ	φ_2	h	k	l	C_1	C_2				
1	319.95	26.97	53.28	0.364	0.271	0.891	37.843	16.720	-4.4	11.7	110	1.47
2	109.61	12.98	36.20	0.133	0.181	0.974	19.150	7.246	-2.64	9.76	23	1.96
3	39.11	49.28	48.56	0.568	0.502	0.652	57.454	41.237	-7.74	12.23	50	1.24
4	111.47	13.86	34.33	0.135	0.198	0.971	20.596	7.508	-2.64	9.98	76	1.89
5	39.61	48.99	47.94	0.560	0.506	0.656	57.020	41.308	-7.56	12.4	31	1.33
6	115.27	12.08	30.79	0.107	0.180	0.978	18.296	6.050	-2.9	12.14	11	2.09
7	1.33	41.82	13.90	0.160	0.647	0.745	60.227	11.831	-4.31	13.1	19	1.29
8	114.44	14.15	31.33	0.127	0.209	0.970	21.298	7.227	-2.55	9.67	69	1.96
9	1.37	41.79	14.15	0.163	0.646	0.746	60.136	12.035	-4.93	12.05	24	1
10	112.49	14.49	33.12	0.137	0.210	0.968	21.623	7.689	-2.46	9.72	36	1.99
11	1.72	41.40	13.40	0.153	0.643	0.750	59.957	11.250	-4.57	12.31	15	1.23
12	224.07	44.12	13.89	0.167	0.676	0.718	62.149	12.775	-3.08	14.95	143	1.28
13	345.07	15.06	23.58	0.104	0.238	0.966	23.232	6.412	-2.95	11.39	16	1.68
14	350.47	15.67	17.77	0.082	0.257	0.963	24.431	5.615	-2.81	10.99	6	1.85
15	299.36	38.64	64.58	0.564	0.268	0.781	54.154	19.407	-6.6	12.66	62	1.37
16	82.86	37.70	88.81	0.611	0.013	0.791	56.670	2.413	-2.81	13.81	13	1.07
17	298.91	38.78	64.53	0.565	0.269	0.780	54.279	19.536	-5.72	11.21	26	1.31
18	320.15	27.09	53.19	0.365	0.273	0.890	37.968	16.842	-4.49	12.23	238	1.37
19	101.02	45.43	75.22	0.689	0.182	0.702	62.815	14.245	-3.08	15.13	9	1.19
20	180.98	35.48	44.34	0.406	0.415	0.814	44.770	27.115	-4.22	12.05	24	1.91
21	274.85	32.52	52.26	0.425	0.329	0.843	44.025	21.643	-5.01	12.93	48	1.51
22	0.35	41.36	15.32	0.175	0.637	0.751	59.440	12.868	-5.36	13.02	87	1.18
23	116.09	12.93	29.75	0.111	0.194	0.975	19.631	6.354	-2.9	10.55	43	1.75
24	267.40	39.32	7.60	0.084	0.628	0.774	58.818	6.203	-2.29	14.86	33	1.21
25	350.60	38.20	80.50	0.610	0.102	0.786	57.191	7.469	-4	13.94	28	1.51
26	106.63	41.44	44.91	0.467	0.469	0.750	49.941	33.787	-6.2	12.97	28	1.1
27	225.97	18.88	74.34	0.312	0.087	0.946	29.388	6.228	-2.55	10.55	53	2.03
28	114.07	11.85	32.33	0.110	0.174	0.979	17.844	6.117	-2.81	8.22	2	1.73
29	312.45	47.30	47.80	0.544	0.494	0.678	55.755	39.373	-7.3	12.58	35	1.21
30	226.16	19.19	74.00	0.316	0.091	0.944	29.847	6.405	-2.73	11.17	96	2.19
31	134.78	30.49	43.72	0.351	0.367	0.862	39.877	22.031	-3.56	12.4	58	2.26
32	103.04	43.83	44.92	0.489	0.490	0.721	51.902	36.561	-6.6	12.75	110	3.48
33	281.27	31.37	44.47	0.365	0.371	0.854	40.562	23.098	-4.31	13.46	105	1.58
34	168.69	46.02	43.21	0.493	0.524	0.694	54.373	38.389	-5.72	12.49	33	1.06
35	30.30	22.21	16.25	0.106	0.363	0.926	34.371	7.410	-3.17	12.23	81	1.78
36	60.64	35.82	20.95	0.209	0.547	0.811	52.259	14.689	-5.28	13.37	21	1.06
37	267.57	39.37	7.25	0.080	0.629	0.773	58.922	5.953	-2.46	17.5	11	1.03
38	321.24	37.79	37.01	0.369	0.489	0.790	49.722	26.123	-6.6	12.93	70	1.45
39	71.17	43.36	8.32	0.099	0.679	0.727	63.023	7.372	-5.45	13.54	19	1.13
40	134.54	30.97	43.92	0.357	0.371	0.857	40.326	22.543	-3.87	12.58	79	2.11
41	343.55	28.85	49.64	0.368	0.312	0.876	39.097	19.464	-5.01	13.9	147	1.46
42	134.02	30.86	45.15	0.364	0.362	0.858	39.892	22.734	-3.74	12.49	10	1.88
43	71.49	44.05	8.15	0.099	0.688	0.719	63.642	7.419	-5.45	15.17	62	0.98
44	173.42	18.39	38.92	0.198	0.245	0.949	26.310	11.128	-2.9	10.99	47	2.04
45	8.56	36.59	60.14	0.517	0.297	0.803	50.798	20.915	-5.45	13.02	8	1.22
46	310.65	20.82	48.75	0.267	0.234	0.935	29.076	13.332	-3.87	13.1	43	1.83
47	49.68	43.49	82.55	0.682	0.089	0.725	63.357	6.614	-2.99	18.12	86	0.99
48	99.52	42.21	45.84	0.482	0.468	0.741	50.915	34.357	-6.24	12.84	3	1
49	206.48	43.63	2.28	0.027	0.689	0.724	64.658	2.030	-2.29	12.23	9	1.45
50	51.44	31.16	1.50	0.014	0.517	0.856	47.360	3.302	-2.64	10.77	46	1.51

51	345.40	40.65	15.09	0.170	0.629	0.759	58.815	12.397	-4.66	12.66	3	0.71
52	68.22	36.03	87.42	0.588	0.026	0.809	54.462	3.336	-2.51	10.91	8	1.02
53	185.12	32.16	14.06	0.129	0.516	0.847	48.793	9.242	-2.73	9.5	23	1.78
54	228.43	45.40	24.63	0.297	0.647	0.702	60.063	23.392	-4.93	12.23	307	1.44
55	314.88	32.84	3.22	0.030	0.541	0.840	49.961	3.908	-2.37	12.58	1073	1.44
56	189.68	34.49	85.30	0.564	0.046	0.824	52.412	4.517	-2.73	9.63	82	2.19
57	227.71	45.23	25.63	0.307	0.640	0.704	59.662	24.175	-4.84	12.31	302	1.4
58	47.74	40.94	64.07	0.589	0.287	0.755	56.179	21.364	-5.36	12.44	14	1.2
59	294.06	19.44	54.49	0.271	0.193	0.943	28.281	11.122	-3.25	9.85	87	1.99
60	149.13	41.00	52.48	0.520	0.400	0.755	52.505	29.585	-4.66	13.41	94	1.32
61	148.34	41.20	53.39	0.529	0.393	0.752	53.006	29.230	-4.05	13.81	5	1.33
62	322.33	38.73	0.18	0.002	0.626	0.780	57.963	1.736	-1.32	9.67	1	1.13
63	243.54	44.33	9.97	0.121	0.688	0.715	63.369	9.216	-2.51	14.03	2	1.56
64	322.31	38.72	0.09	0.001	0.626	0.780	57.934	1.697	-2.9	12.66	11	1.36
65	244.34	44.46	9.74	0.118	0.690	0.714	63.527	9.051	-2.64	14.2	82	1.01
66	5.06	39.13	83.52	0.627	0.071	0.776	58.699	5.404	-3.08	13.9	9	1.04
67	86.81	31.08	1.29	0.012	0.516	0.856	47.219	3.227	-2.64	11.52	43	1.82
68	16.60	32.51	39.28	0.340	0.416	0.843	43.523	22.275	-5.94	13.72	6	1.2
69	189.31	42.80	48.01	0.505	0.455	0.734	52.323	34.017	-5.19	13.85	28	1.27
70	45.30	41.66	64.72	0.601	0.284	0.747	56.998	21.349	-5.63	12.66	87	1.19
71	321.01	33.57	60.34	0.481	0.274	0.833	47.546	18.572	-5.5	13.02	36	1.48

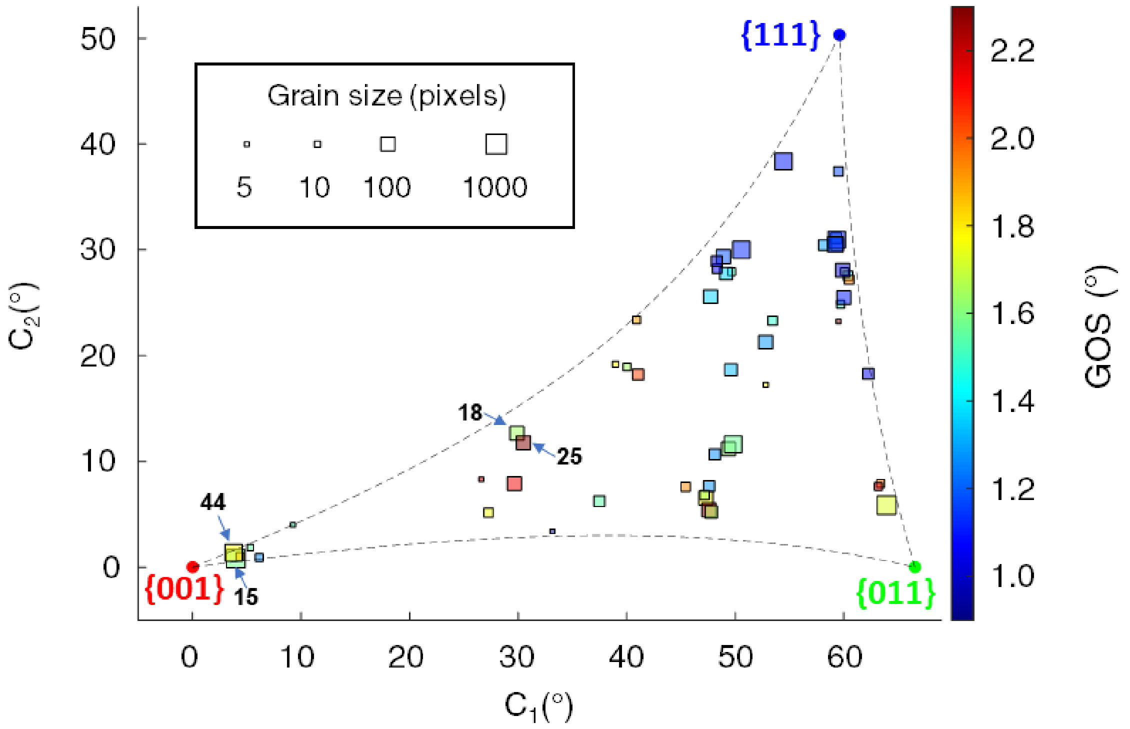

Figure S11. Correlation plot for the grain orientation spread (GOS) for the inhibitor-free case.

REFERENCES

1. Platzman, I.; Brener, R.; Haick, H.; Tannenbaum, R., Oxidation of Polycrystalline Copper Thin Films at Ambient Conditions. *J. Phys. Chem. C* **2008,** *112* (4), 1101-1108.
2. Boggio, J. E., The room temperature oxidation of Cu (111): Pressure effects. *J. Chem. Phys.* **1979,** *70* (11), 5054-5058.
3. Iijima, J.; Lim, J. W.; Hong, S. H.; Suzuki, S.; Mimura, K.; Isshiki, M., Native oxidation of ultra high purity Cu bulk and thin films. *Appl. Surf. Sci.* **2006,** *253* (5), 2825-2829.
4. Gattinoni, C.; Michaelides, A., Atomistic details of oxide surfaces and surface oxidation: the example of copper and its oxides. *Surf. Sci. Rep.* **2015,** *70* (3), 424-447.
5. Nakayama, S.; Kaji, T.; Shibata, M.; Notoya, T.; Osakai, T., Which Is Easier to Reduce, Cu_2O or CuO? *J. Electrochem. Soc.* **2007,** *154* (1), C1.
6. Daviddi, E.; Shkirskiy, V.; Kirkman, P. M.; Robin, M. P.; Bentley, C. L.; Unwin, P. R., Nanoscale electrochemistry in a copper/aqueous/oil three-phase system: surface structure–activity-corrosion potential relationships. *Chem. Sci.* **2021,** *12* (8), 3055-3069.
